# Skeletal muscle p53‐depletion uncovers a mechanism of fuel usage suppression that enables efficient energy conservation

**DOI:** 10.1002/jcsm.13529

**Published:** 2024-07-15

**Authors:** Georgia Lenihan‐Geels, Francisco Garcia Carrizo, Marina Leer, Sabrina Gohlke, Moritz Oster, Sophie Pöhle‐Kronawitter, Christiane Ott, Alexandra Chadt, Isabel N. Reinisch, Markus Galhuber, Chen Li, Wenke Jonas, Markus Jähnert, Susanne Klaus, Hadi Al‐Hasani, Tilman Grune, Annette Schürmann, Tobias Madl, Andreas Prokesch, Michael Schupp, Tim J. Schulz

**Affiliations:** ^1^ Department of Adipocyte Development and Nutrition German Institute of Human Nutrition Potsdam‐Rehbrücke (DIfE) Nuthetal Germany; ^2^ German Center for Diabetes Research (DZD) München‐Neuherberg Germany; ^3^ Charité Universitätsmedizin Berlin, corporate member of Freie Universität Berlin and Humboldt‐Universität zu Berlin Institute of Pharmacology, Max Rubner Center (MRC) for Cardiovascular‐Metabolic‐Renal Research Berlin Germany; ^4^ Department of Molecular Toxicology German Institute of Human Nutrition Potsdam‐Rehbrücke Nuthetal Germany; ^5^ Institute for Clinical Biochemistry and Pathobiochemistry, German Diabetes Center (DDZ) Leibniz Center for Diabetes Research at Heinrich Heine University Düsseldorf Germany; ^6^ Gottfried Schatz Research Center for Cell Signaling, Metabolism and Aging, Division of Cell Biology, Histology and Embryology Medical University of Graz Graz Austria; ^7^ Department of Experimental Diabetology German Institute of Human Nutrition Potsdam‐Rehbrücke Nuthetal Germany; ^8^ Department Physiology of Energy Metabolism German Institute of Human Nutrition Potsdam‐Rehbrücke Nuthetal Germany; ^9^ Gottfried Schatz Research Center for Cell Signaling, Metabolism and Aging, Division of Molecular Biology and Biochemistry Medical University of Graz Graz Austria; ^10^ BioTechMed‐Graz Graz Austria; ^11^ Institute of Nutritional Science University of Potsdam, Potsdam‐Rehbrücke Nuthetal Germany

**Keywords:** Energy conservation, p53, Metabolic efficiency, Metabolic homeostasis, Metabolism, Skeletal muscle

## Abstract

**Background:**

The ability of skeletal muscle to respond adequately to changes in nutrient availability, known as metabolic flexibility, is essential for the maintenance of metabolic health and loss of flexibility contributes to the development of diabetes and obesity. The tumour suppressor protein, p53, has been linked to the control of energy metabolism. We assessed its role in the acute control of nutrient allocation in skeletal muscle in the context of limited nutrient availability.

**Methods:**

A mouse model with inducible deletion of the p53‐encoding gene, *Trp53*, in skeletal muscle was generated using the Cre‐loxP‐system. A detailed analysis of nutrient metabolism in mice with control and knockout genotypes was performed under *ad libitum* fed and fasting conditions and in exercised mice.

**Results:**

Acute deletion of p53 in myofibres of mice activated catabolic nutrient usage pathways even under *ad libitum* fed conditions, resulting in significantly increased overall energy expenditure (+10.6%; *P* = 0.0385) and a severe nutrient deficit in muscle characterized by depleted intramuscular glucose and glycogen levels (−62,0%; *P* < 0.0001 and −52.7%; *P* < 0.0001, respectively). This was accompanied by changes in marker gene expression patterns of circadian rhythmicity and hyperactivity (+57.4%; *P* = 0.0068). These metabolic changes occurred acutely, within 2–3 days after deletion of *Trp53* was initiated, suggesting a rapid adaptive response to loss of p53, which resulted in a transient increase in lactate release to the circulation (+46.6%; *P* = 0.0115) from non‐exercised muscle as a result of elevated carbohydrate mobilization. Conversely, an impairment of proteostasis and amino acid metabolism was observed in knockout mice during fasting. During endurance exercise testing, mice with acute, muscle‐specific *Trp53* inactivation displayed an early exhaustion phenotype with a premature shift in fuel usage and reductions in multiple performance parameters, including a significantly reduced running time and distance (−13.8%; *P* = 0.049 and −22.2%; *P* = 0.0384, respectively).

**Conclusions:**

These findings suggest that efficient nutrient conservation is a key element of normal metabolic homeostasis that is sustained by p53. The homeostatic state in metabolic tissues is actively maintained to coordinate efficient energy conservation and metabolic flexibility towards nutrient stress. The acute deletion of *Trp53* unlocks mechanisms that suppress the activity of nutrient catabolic pathways, causing substantial loss of intramuscular energy stores, which contributes to a fasting‐like state in muscle tissue. Altogether, these findings uncover a novel function of p53 in the short‐term regulation of nutrient metabolism in skeletal muscle and show that p53 serves to maintain metabolic homeostasis and efficient energy conservation.

## Introduction

Impaired glucose homeostasis in muscle promotes systemic metabolic inflexibility and is frequently observed in individuals with obesity and the metabolic syndrome.^1^ The transcription factor p53 is a cell cycle regulator and tumour suppressor and has also been implicated in metabolic control. A short‐lived protein under basal conditions, p53 is stabilized by many stressors, including DNA damage, hypoxia, and low‐nutrient availability.^2^, ^3^, ^4^, ^5^, ^6^ In response to exercise, p53 localizes to both nuclei and mitochondria in myofibres.^7^, ^8^, ^9^ Several studies on p53 function in skeletal muscle have focused on training‐related adaptations and exercise performance, sometimes yielding contradictive results.^8^, ^10^, ^11^ Murine p53 loss‐of‐function models show impaired mitochondrial function in liver and skeletal muscle and reduced exercise capacity.^11^, ^12^ Conversely, congenital skeletal muscle‐specific ablation of p53 does not appear to affect muscle mitochondrial oxidative phosphorylation or nutrient metabolism, suggesting that myofibres are able to adapt to the absence of p53 to sustain normal mitochondrial function.^13^ A separate study found that skeletal muscle‐specific p53 knockout mice had impaired exercise performance due to lower mitochondrial oxidative capacity, although these defects were ameliorated following a six‐week training programme.^10^ A transcriptional p53 signalling programme is induced upon short‐term fasting in skeletal muscle, adipose tissue, and liver, suggesting that p53 is a key element of the response to nutrient deprivation across multiple metabolic organs.^5^ We previously showed that p53 regulates hepatic gluconeogenesis and glycogen metabolism in the fasted state, and liver‐specific deletion of p53 attenuates amino acid catabolism, leading to hypoglycaemia in fasted mice.^4^, ^14^ In summary, p53‐associated signalling is activated in response to changes in nutrient availability in several tissues. However, how p53 responds to acute changes in nutrient availability to coordinate appropriate metabolic responses in skeletal muscle has not been investigated. Here we use an acutely inducible mouse model with myofibre‐specific *Trp53* deletion (p53MKO), thereby preventing the long‐term adaptive effects associated with other genetic knockout models that may have a predisposition to tumour formation. Our findings address a novel function of p53 in the short‐term regulation of nutrient metabolism in skeletal muscle, showing that p53 serves to maintain metabolic homeostasis and efficient energy conservation.

## Methods

All animal experiments were performed in accordance with regulations of the ethics committee for animal welfare of the State Office of Environment, Health, and Consumer Protection (State of Brandenburg, Germany). p53‐deficient mice were generated with the Cre‐loxP technology by intercrossing floxed *Trp53* mice with animals expressing a tamoxifen‐inducible Cre‐recombinase under control of the myofibre‐specific *Acta1*‐promoter (p53MKO). p53MKO mice were compared with loxP homozygous animals without Cre‐allele (LOX). p53MKO and LOX mice were treated with tamoxifen for 5 days, resulting in*Trp53* gene inactivation in p53MKO. Experiments were performed on male p53MKO and LOX‐control mice aged approximately 15–20 weeks. Mice were subjected to indirect calorimetry in the fed state, during fasting and treadmill running. Skeletal muscle samples were isolated for mRNA and protein expression analyses, for metabolite analysis by NMR spectroscopy, and biochemical enzyme activity assays.

## Results

### p53 signalling is induced upon short‐term fasting in skeletal muscle

**Figure 1:**
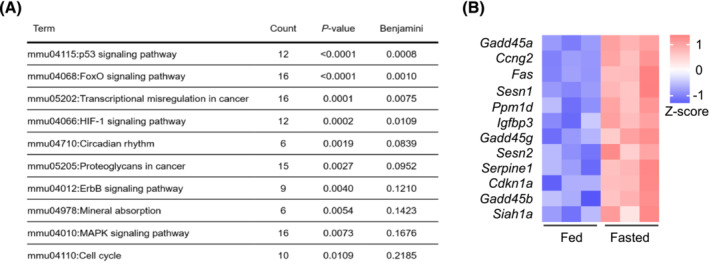
p53 signalling is induced following 16 h fasting in quadriceps muscle. (A) KEGG pathway analysis from quadriceps muscle RNAseq analysis comparing differentially expressed genes (*P* < 0.05, log‐fold change [LFC] > 1) in control versus 16‐h fasted, wild‐type C57BL/6J mice (*n* = 3 per group). Counts depicts the number of genes in each KEGG term. (B) Genes upregulated in quadriceps of 16‐h fasted mice compared with control from KEGG term p53 signalling pathway from RNAseq analysis.

To assess transcriptional responses to the metabolic challenge of fasting in skeletal muscle, C57BL/6J wild‐type mice were subjected to 16 h of food withdrawal and compared with animals maintained with *ad libitum* food supply. Functional annotation of the transcriptomes of quadriceps muscle revealed the p53 signalling pathway as the most significantly upregulated KEGG term, suggesting an involvement of p53 in the fasting response in skeletal muscle (Figure 1A; Tables S1–S4). Genes derived from the p53 signalling term could be broadly categorized into two areas, cell‐cycle regulation (e.g., *Ccng2* and *Gadd45a*) and cellular stress response (e.g., *Sesn1*, *Sesn2* and *Siah1a*) (Figure 1B). Expression of a panel of known fasting‐regulated p53‐target genes was measured in several skeletal muscles and in liver. Consistent with our previous analyses^4^, ^5^ and further supporting the hypothesis that p53 is involved in the fasting response, p53‐linked transcripts, *Cdkn1a*, *Ddit4*, *Lpin1*, and *Sesn2*, were upregulated after 16 h of fasting in skeletal muscles and liver (Figure S1A–D).

### Acute *Trp53* deletion in myofibres initiates a hyperactivity phenotype in mice

**Figure 2:**
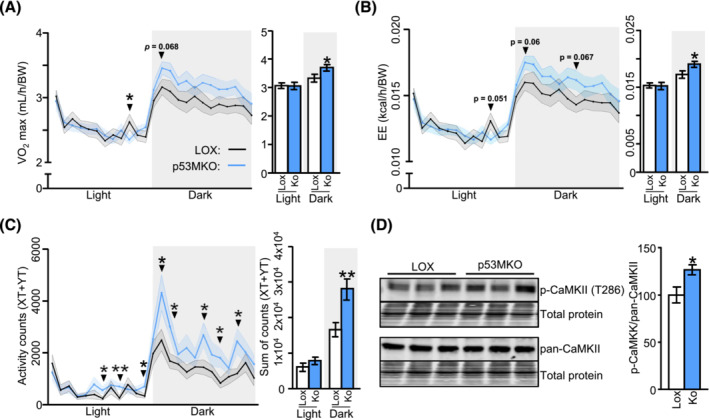
Acute deletion of *Trp53* in myofibres increases energy expenditure and locomotor activity. (A) Oxygen consumption (VO2) and average maximal oxygen consumption (VO2 max; right panel) in light/dark phase of control mice carrying lox‐P elements on both alleles but do not carry a CreERT2‐allele (LOX; black line /white bars) and p53MKO (blue line/blue bars) mice after 5‐day tamoxifen exposure and 2‐day recovery. (B) Energy expenditure (EE) and average maximal energy expenditure (EE max; right panel) in light/dark phase of control and p53MKO mice. (C) Activity counts expressed as XT + YT movements and sum of activity counts (XT + YT; right panel) in light/dark phase in control and p53MKO mice. (D) Protein levels of phosphorylated‐CaMKII (T286) and pan‐CaMKII in quadriceps muscle, with quantification of p‐CaMKII (T286) relative to pan‐CaMKII, normalized to total protein loading. Results presented as mean ± SEM and compared by *t*‐test with Welch's correction, or multiple *t*‐testing (*n* = 14 for A–C; *n* = 8–10 for D); *P < 0.05, **P < 0.01.

To further assess the role of p53 in skeletal muscle, inducible myofibre‐specific *Trp53* knockout mice (p53MKO) were compared with tamoxifen‐treated, floxed control littermates (LOX). All mice were characterized after a short, 48‐h recovery after induction of the knockout to monitor acute changes rather than post‐adaptation processes secondary to those immediately caused by gene deletion (Figure S2A, B). The knockout efficiency in p53MKO animals was approximately 50% in skeletal muscle (Figure S2C), which is consistent with previous reports of conditional muscle‐specific *Trp53* knockout mouse models.^13^ Residual expression of *Trp53* mRNA may derive from non‐myofibre cells within skeletal muscle. Acute p53MKO mice showed no changes in body mass and composition, skeletal muscle mass, expression of fibre type‐specific genes or myofibre sizes (Figures S2D–H). Metabolic characterization by indirect calorimetry showed that acute p53MKO mice displayed similar substrate oxidation patterns, as demonstrated by unchanged respiratory exchange ratios (RER), carbohydrate‐, and lipid oxidation (Figures S3A–F). However, p53MKO mice had increased oxygen consumption (VO2) and energy expenditure (EE) during the active, dark phase compared with LOX‐control mice (Figure 2A,B). This corresponded with significantly higher locomotor activity (Figure 2C), but no changes to plasma corticosterone or cortisol levels (Figure S3G,H). Phosphorylation of Ca2+/calmodulin‐dependent protein kinase II (CaMKII; T286), an active form of CaMKII that is increased in response to contraction in myofibres,^15^ was elevated in p53MKO quadriceps muscle, further supporting the notion of increased activity (Figure 2D). No genotype‐dependent differences were found during 16 h of food withdrawal for RER, VO2, EE, or carbohydrate and lipid oxidation (Figures S3I–M). Taken together, these data establish that mice with short‐term muscle‐specific ablation of *Trp53* exhibit significant increases in EE, which may be explained by increased activity and enhanced substrate usage in skeletal muscle.

### *Trp53*‐gene deletion alters the circadian gene expression profile to resemble a fasting‐like state

**Figure 3:**
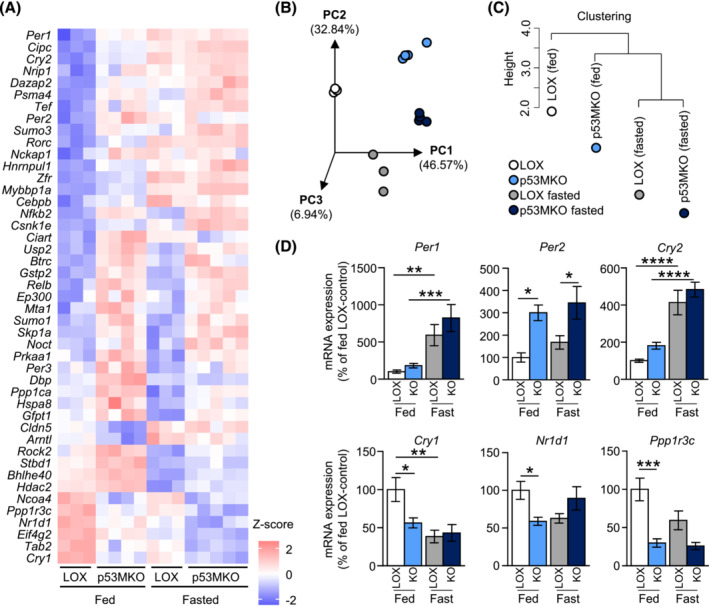
Acute loss of p53 induces a fasting‐like circadian gene expression signature in muscle. (A) Heatmap of circadian gene transcripts altered (*P* < 0.05; presented as *Z*‐scores) by acute *Trp53* deletion in quadriceps in fed and fasted conditions from RNAseq analysis comparing control (LOX) and p53MKO (KO) mice under fed and 16‐h fasted conditions (*n* = 3–5). (B) 3D principal component analysis (PCA) representation and (C) hierarchical clustering analysis of fed control (white/black circles), fed p53MKO (light blue circles), fasted control (grey circles), and fasted p53MKO (dark blue circles) mice based on circadian‐related gene transcripts. (D) mRNA expression of *Per1*, *Per2*, *Cry2*, *Cry1*, *Nr1d1*, and *Ppp1r3c* in quadriceps of p53MKO mice in fed (LOX: White bars; p53MKO blue bars) and 16‐h fasted (LOX: Grey bars; p53MKO: Dark blue bars) conditions. Results presented as mean ± SEM; data in (D) were compared by two‐way ANOVA (*n* = 8–11); *P < 0.05, **P < 0.01, ***P < 0.001, ****P < 0.0001.

To elucidate the mechanisms leading to increased hyperactivity in the p53MKO model, transcriptomes of quadriceps muscle from LOX and p53MKO animals were compared. Functional annotation of differentially expressed genes revealed several nutrient‐related terms that were significantly altered by acute *Trp53* gene deletion in myofibres (Tables S5–S8). Consistent with the observed phenotype of increased activity, p53MKO muscles displayed altered expression of genes regulating skeletal muscle contraction (Tables S7, S8). Our functional annotation also indicated terms related to protein metabolism and insulin and calcium signalling. Several upregulated terms were reminiscent of those earlier identified as fasting‐induced pathways, such as FoxO signalling and MAPK signalling (Table S5; Figure 1A). A more detailed transcriptomic analysis also revealed alterations in the expression of multiple genes related to circadian rhythmicity in p53MKO mice (Figure 3A). Since a circadian gene expression signature also appeared to be enriched by fasting (Figure 1A), a principal component analysis of the marker gene expression profiles from quadriceps muscle of fed versus fasted and LOX‐control versus p53MKO animals was conducted. This analysis revealed a clear separation between all four groups (Figure 3B), but a higher degree of similarity between fed p53MKO muscle and the two fasted groups, as opposed to the fed state in LOX‐control animals (Figure 3C). Accordingly, several core circadian genes, including *Per1*, *Per2*, and *Cry2*, were induced in fed p53MKO and by fasting. Conversely, circadian genes down‐regulated by fasting, *Cry1*, *Nr1d1*, and *Ppp1r3*, were lower expressed in fed p53MKO mice compared with fed control mice (Figure 3D). Taken together, these findings suggest that acute loss of *Trp53* in myofibres affects circadian pathways in skeletal muscle and shifts the transcriptional state towards a fasting‐like profile.

### Acute myofibre‐specific ablation of *Trp53* alters muscle protein catabolism

**FIGURE 4:**
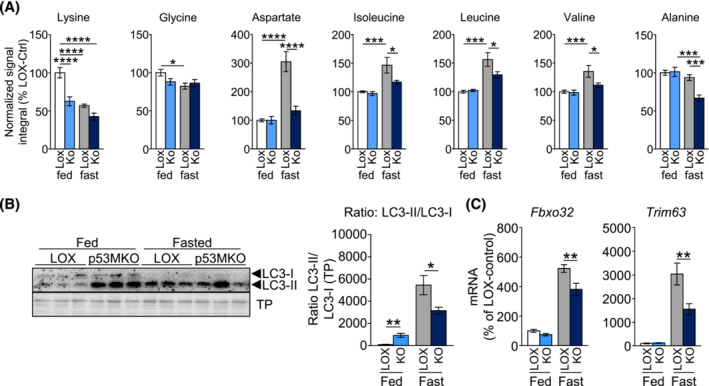
p53MKO mice demonstrate distinct changes in amino acid metabolism under fed and fasted conditions. (A) NMR spectroscopic analysis of intramuscular amino acids lysine, glycine, alanine, aspartate, isoleucine, leucine, valine and alanine in quadriceps of control (LOX) and p53MKO (KO) mice in the fed state (control: white bars; p53MKO: light blue bars) and following 16 h of food withdrawal (control: grey bars; p53MKO: dark blue bars). (B) Representative detection (left panel) of protein levels of LC3‐II and LC3‐I in quadriceps muscle of control and p53MKO mice in the fed state (LOX: white bars; p53MKO: blue bars) and following 16 h of food withdrawal (LOX: grey bars; p53MKO: dark blue bars) and quantification of LC3II:LC3I ratio normalized to total protein (right panel). (C) mRNA expression of *Fbxo32* and *Trim63* in quadriceps muscle under similar conditions. Results presented as mean ± SEM and compared by two‐way ANOVA (*n* = 5–10 for A and B, and *n* = 9–11 for C); *P < 0.05, **P < 0.01, ***P < 0.001, ****P < 0.0001.

The transcriptomic profile of p53MKO animals additionally displayed a concerted shift towards increased protein catabolism, while protein synthesis pathways were collectively downregulated (Figure S4; Tables S5, S6). For instance, genes encoding proteasomal subunits (e.g., *Psmc1* and *Psma2*), E3 ubiquitin‐ligases (e.g., *Cul5* and *Herc4*), and ubiquitin‐conjugating enzymes (e.g., *Ube2g1*, and *Ube2h*) were induced in quadriceps of p53MKO mice in *ad libitum* fed conditions (Figure S4A). This coincided with lower expression of protein synthesis markers, such as genes of the mammalian target of rapamycin (mTOR) and ribosomal pathways (Figure S4B). Paralleling the transcriptomic changes, a metabolomic analysis of quadriceps and gastrocnemius identified significant changes in intramuscular amino acid levels. Consistent with the notion of a fasting‐like phenotype in muscles of p53MKO mice in the fed state, lysine was significantly reduced in fed p53MKO animals compared with LOX‐controls and was also reduced upon fasting in both genotypes (Figure 4A [quadriceps] and S5A [gastrocnemius]). Aside from a fasting‐induced decrease, no genotype‐dependent differences were found in glycine levels. Importantly, fasting resulted in a significant increase in the amino acid aspartate and the branched‐chain amino acids (BCAAs) isoleucine, leucine, and valine, which are considered essential substrates for hepatic gluconeogenesis during fasting,^16^ and this effect was absent in p53MKO mice (Figures 4A and S5A). Alanine was significantly increased in fed p53MKO animals in gastrocnemius but not quadriceps muscle, and was significantly reduced upon fasting in both muscles of p53MKO mice, suggesting that alanine may serve as a source of energy in knockout mice during fasting (Figures 4A and S5A). In line with this notion, expression of the transaminase gene *Gpt2* was elevated in fed p53MKO mice (Figure S5B).

Consistent with increased protein breakdown under fed conditions, p53MKO animals exhibited increased levels of autophagosome marker, LC3‐II, reaching levels in fed p53MKO mice that were observed in the fasted state of both genotypes (Figure 4B). As LC3‐I levels were similar in fed mice of both genotypes, this resulted in a significant increase in the LC3‐II/LC3‐I protein ratio in fed p53MKO mice. This effect was reversed under fasting conditions, which showed a significant reduction of the ratio in p53MKO, suggesting that acute loss of p53 results in impaired protein mobilization only during fasting (Figure 4B). In line with potentially increased autophagy in fed p53MKO mice, several autophagy genes were induced (Figure S5C).^17^ In addition, quadriceps from p53MKO animals showed significantly blunted expression of muscle atrophy genes, *Fbxo32* and *Trim63*, during fasting (Figure 4C). However, neither LOX control nor p53MKO mice exhibited altered levels of K48‐linked or K63‐linked poly‐ubiquitination or proteasome activity in fed or fasted states (Figure S5D–F). These findings altogether suggest that fed p53MKO mice feature a mild increase of proteolytic markers, whereas fasted p53MKO mice display a blunted induction of the well‐established increase in proteolysis during fasting.

### Acute p53 deletion in skeletal muscle promotes a transient increase in carbohydrate oxidation and creates an intramuscular glucose deficit

**Figure 5:**
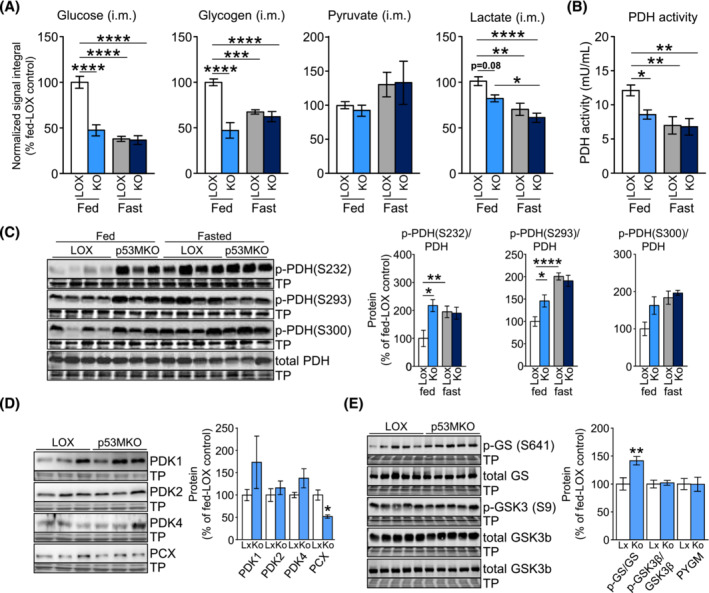
Acute *Trp53* deletion results in depleted intramuscular glucose and glycogen stores. (A) NMR spectroscopic analysis of intramuscular glucose, glycogen, pyruvate, and lactate levels in quadriceps muscle from control (LOX) and p53MKO (KO) mice during fed conditions (LOX: white bars; p53MKO: light blue bars) and following 16 h of food withdrawal (LOX: grey bars; p53MKO: dark blue bars). (B) Pyruvate dehydrogenase (PDH) activity in quadriceps of control and p53MKO mice under fed and fasted conditions. (C) Representative immunodetections (left panels) and quantifications (right panels) of phosphorylated PDH (p‐PDH at residues: S232, S293, and S300) and total PDH in quadriceps muscle under fed and fasted conditions and normalized to total PDH and total protein loading. (D) Representative immunodetections (left panels) and quantifications (right panels) of pyruvate dehydrogenase kinase isoforms (PDK1, PDK2, and PDK4) and pyruvate carboxylase (PCX) in quadriceps muscle under fed and fasted conditions and normalized to total protein loading. (E) Representative immunodetections (left panels) and quantifications (right panels) of glycogen synthase (GS) phosphorylation (p‐GS at S641), total GS, glycogen synthase kinase 3‐beta (GSK3‐beta) phosphorylation (p‐GHSK3b at S9), total GSK3‐beta, and total glycogen phosphorylase (PYGM) in quadriceps muscle under fed and fasted conditions and normalized to total corresponding protein (for p‐GS, p‐GSK3‐beta) and total protein loading. Results presented as mean ± SEM; data in (A)–(C) compared by two‐way ANOVA (*n* = 8–10); data in (C) and (E) compared by Mann–Whitney test (*n* = 7–11); *P < 0.05, **P < 0.01, ***P < 0.001, ****P < 0.0001.

Because several amino acids were found to be altered in p53MKO mice with and without fasting, we also examined other nutrients, including those linked to glucose homeostasis. Significantly lower concentrations of glycogen and glucose in both muscles, but no differences in pyruvate, were observed (Figures 5A and S6A). Generated by glycolysis, pyruvate can be converted to lactate or acetyl‐coenzyme A (acetyl‐CoA). Lactate levels showed a trend for reduction in quadriceps and were unchanged in gastrocnemius muscle of fed p53MKO (Figures 5A and S6A). The activity of the pyruvate dehydrogenase (PDH) complex, which catalyses the oxidation of pyruvate to form acetyl‐CoA and carbon dioxide, is a marker of subsequent mitochondrial oxidation of pyruvate generated in glycolysis.^18^ Unexpectedly, both muscles from fed p53MKO mice presented significantly lower PDH activities, which decreased to levels seen in fasted animals in both genotypes (Figures 5B and S6B). This was accompanied by significantly increased inhibitory phosphorylation of PDH at several phosphorylation sites in quadriceps muscle of fed p53MKO mice (Figure 5C). PDH activity is regulated by different factors, including the inhibitory pyruvate dehydrogenase kinases (PDKs),^18^ but no differences in the expression of several PDH regulatory genes (*Pdk2*, *Pdk4*, *Pdp1*, and *Pdha1*) were found in quadriceps or gastrocnemius of p53MKO animals (Figure S6C–D). Likewise, PDK protein levels appeared largely unchanged in p53MKO mice (Figure 5D). Pyruvate carboxylase (PCX), which promotes anaplerotic contribution of pyruvate to the citric acid cycle as oxaloacetate, was significantly downregulated (Figure 5D).

Citrate synthase activity and oxidative phosphorylation complexes were unchanged, ruling out a generalized mitochondrial defect in p53MKO mice (Figure S6E–G). Expression of genes of mitochondrial fatty acid uptake and oxidation pathways were induced in quadriceps muscle of p53MKO animals in the fed and acutely fasted states, suggesting acetyl‐CoA for tricarboxylic acid cycling may be derived from lipid sources (Figure S6H). Lastly, the decline of intramuscular glycogen reservoirs corresponded with significantly higher inhibitory phosphorylation of glycogen synthase (GS), while phosphorylated glycogen synthase kinase 3‐beta (GSK3‐beta) and muscle glycogen phosphorylase (PYGM) levels were unchanged (Figure 5E).

### Acute inactivation of p53 in skeletal muscle induces a transient spike in glucose utilization

**Figure 6:**
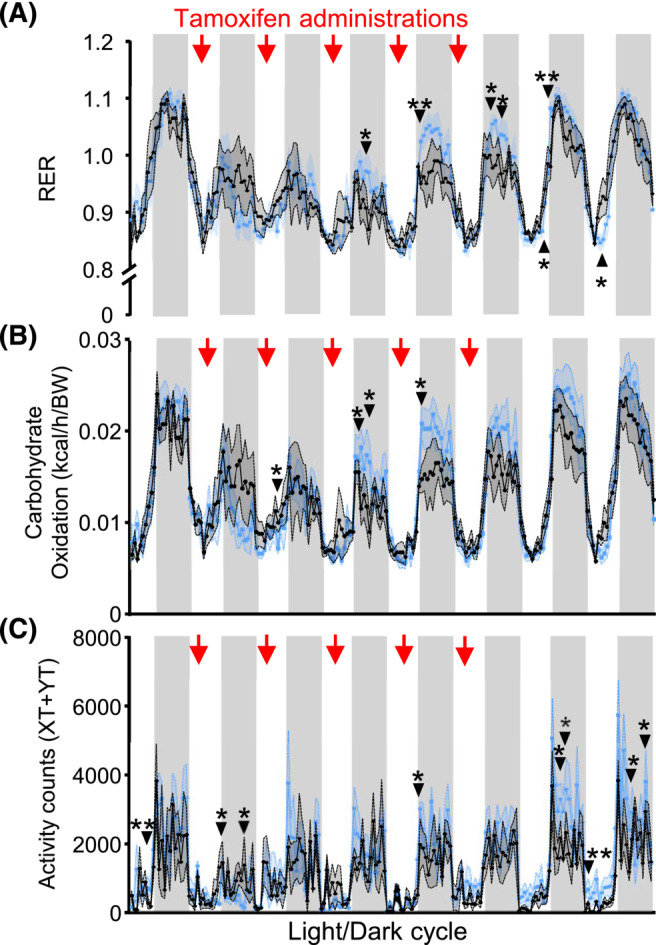
Acute *Trp53* deletion in myofibres leads to rapid and transient metabolic adaptations. (A) Respiratory exchange ratio (RER), (B) carbohydrate oxidation, (C) activity counts measured as XT + YT movements in control (black line) and p53MKO (blue line) animals with *ad libitum* food access during 8 days' tracing energy metabolism changes during induced *Trp53* inactivation. Red arrows indicate days of tamoxifen administration. Results presented as mean ± SEM and were compared by multiple *t*‐tests (*n* = 4–5); *P < 0.05, **P < 0.01.

Based on these observations and supportive literature from cancer cells,^19^, ^20^ we postulated that p53 may act to repress glucose oxidation in skeletal muscle under normal physiological conditions to help sustain efficient energy conservation. Because the analysis of systemic energy metabolism indicated no changes to fuel oxidation between LOX‐control and p53MKO mice (Figure S3A–F), metabolic changes leading to glycogen and glucose depletion in p53MKO muscle may develop during the earliest stages of *Trp53* gene inactivation, which would not have been visible in our later analyses. A cohort of LOX‐control and p53MKO animals was therefore subjected to indirect calorimetry throughout tamoxifen administration to elucidate whether nutrient flux changes occur during the initial stages of CreERT2‐mediated *Trp53* gene deletion. As hypothesized, p53MKO mice displayed a transient increase of RER values during the dark phases following the third day of tamoxifen administration, which is indicative of increased carbohydrate usage for oxidative phosphorylation (Figure 6A). This temporary phenotype was not sustained during the final phases of calorimetry and is therefore consistent with the original assessment of energy metabolism 6 days after the initial tamoxifen administration (Figure S3A). Agreeing with the transient changes of the RER, the systemic carbohydrate oxidation index was temporarily increased during the same dark phases (Figure 6B). The increased expression of fatty acid metabolism genes in quadriceps of p53MKO mice (Figure S6H) was further corroborated by a mild induction of lipid oxidation during light phases of later time points (Figure S7A). This observation could indicate the time point at which intramuscular glycogen stores are exhausted following acute loss of *Trp53*, thereby enforcing utilization of alternative fuel substrates. Locomotor activity and energy expenditure began to increase significantly in p53MKO animals in dark phases at the end of the tamoxifen administration protocol (Figures 6C and S7B). Because locomotion of p53MKO mice is heightened only when intramuscular energy substrates were likely depleted, this could represent an increase in foraging behaviour characteristic of fasted animals.^21^ However, no differences in food intake were observed between LOX control and p53MKO mice (Figure S7C). No differences were found in gluconeogenic gene expression, hepatic triglycerides or glycogen stores, but a mild, non‐significant decrease in epididymal and subcutaneous white adipose tissue weight was observed in fed p53MKO animals (Figure S8A–E).

### Transient glucose utilization in skeletal muscle occurs through anaerobic lactate production and release upon p53 inactivation

**Figure 7:**
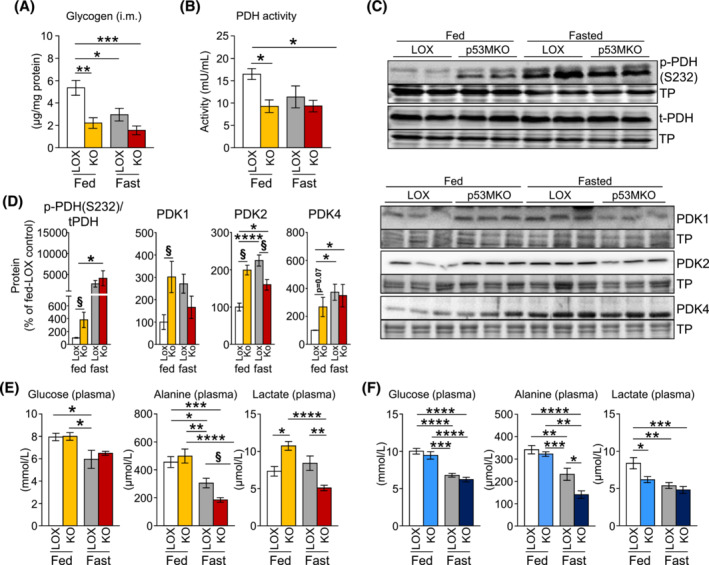
Anaerobic glucose breakdown occurs in response to elevated glucose mobilization following *Trp53* inactivation. (A) Intramuscular glycogen levels, measured by a biochemical assay, in quadriceps muscle from control (LOX) and p53MKO (KO) mice during fed conditions (LOX: white bars; p53MKO: yellow bars) and following 4 h of food withdrawal (4 pm to 8 pm; LOX: Grey bars; p53MKO: orange bars) in mice directly after an abbreviated course of short‐term (3‐day) tamoxifen administration. (B) Pyruvate dehydrogenase (PDH) activity in quadriceps of control and p53MKO mice under fed and fasted conditions after short‐term tamoxifen. (C) Representative immunodetections and (D) quantifications of phosphorylated PDH (p‐PDH at S232), total PDH (t‐PDH), pyruvate dehydrogenase kinase isoforms (PDK1, PDK2, and PDK4) in quadriceps muscle under fed and fasted conditions and normalized to total corresponding protein (for p‐PDH) and total protein (TP) loading (conditions as described in panel A). (E) Plasma glucose, alanine, and lactate measured in plasma in fed and fast conditions in mice directly after an abbreviated course of short‐term (3‐day) tamoxifen administration. (F) Plasma glucose, alanine, and lactate measured in plasma of LOX and p53MKO mice during fed conditions (LOX: white bars; p53MKO: light blue bars) and following 16 h of food withdrawal (LOX: grey bars; p53MKO: dark blue bars) after receiving the full course of 5‐day tamoxifen and 2‐day recovery before organ collection. Results presented as mean ± SEM; data were compared by two‐way ANOVA (A–E: *N* = 3–5; F: *N* = 8–10); *P < 0.05, **P < 0.01, ***P < 0.001, ****P < 0.0001.

To further investigate the hypothesis that early metabolic adaptations may occur during the initial stages of *Trp53* gene deletion, a cohort of LOX control and p53MKO animals was subjected to 3 days of tamoxifen administration followed by 4 h of food withdrawal at the end of the light phase and compared with animals maintained with *ad libitum* food supply. Glycogen levels in quadriceps muscle were significantly reduced in p53MKO mice under fed conditions, decreasing to levels seen in both genotypes after a short 4‐h fast (Figure 7A). Similarly, PDH activity was significantly reduced in fed p53MKO, down to fasting levels, and was accompanied by increased inhibitory phosphorylation at serine 232 and a similar reduction in PCX protein (Figures 7B–D and S8F). Importantly, and diverging from the observations in the full (7‐day) time‐course experiment, we observed a significant increase in the protein levels of PDK‐1, ‐2, and ‐4 in quadriceps muscle of fed p53MKO mice, similar to the increment seen in both genotypes after the 4‐h fast (Figure 7C,D). Altogether, this suggests that changes in nutrient metabolism are among the earliest consequences of p53 deletion and consistent with an early reduction of PDH activity. To verify whether this would indeed lead to the preferential metabolization of surplus glucose via anaerobic glycolysis, we examined plasma metabolites in both experiments, i.e. after the short‐term (3‐day) and full (7‐day) time‐courses. Circulating glucose and alanine levels were reduced in response to fasting in both genotypes and alanine levels dropped significantly further in fasted p53MKO only, to some extent corroborating our observation that loss of p53 in fasted muscle may result in attenuated protein mobilization (Figure 7E,F). In line with our previous data, we also detected significant reductions of the LC3‐II over LC3‐I ratio and lower expression of atrophy genes *Fbxo32* and *Trim63* in the short‐term experiment (Figure S9). Circulating lipids remained unchanged in either experiment, and insulin was unchanged in the full‐time course experiment (Figure S10). Importantly, we found circulating lactate to be significantly increased in fed p53MKO in the short‐term experiment, while lactate was significantly reduced at later time points, as seen in the full (7‐day) time‐course experiment (Figure 7E,F). These data suggest that the early p53‐related unblocking of glucose mobilization in skeletal muscle mainly results in anaerobic glucose utilization and subsequent release as lactate.

### Exercise capacity and fuel selection is impaired in mice with acute inactivation of *Trp53*

**FIGURE 8:**
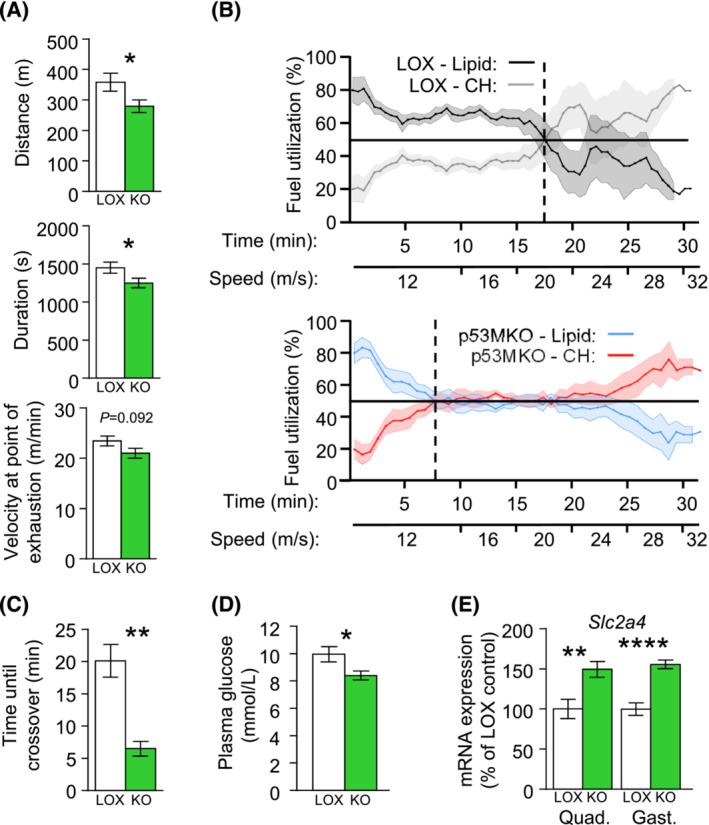
p53MKO mice exhibit reduced exercise capacity and metabolic inflexibility. (A) Total distance of run, duration of run, and final velocity at point of exhaustion comparing control (LOX; white bars) and p53MKO (KO; green bars) mice undergoing an acute endurance treadmill running bout until exhaustion. (B) Percent fuel utilization of control animals (upper panel), depicting percentage of lipid (black line) and CH oxidation (grey line), and p53MKO animals (lower panel; lipid: blue line; CH: red line). The point of crossover, at which lipid and CH fuel utilization are equal, is represented by the vertical dashed lines. (C) Time at sustained first crossover point in control (white bar) and p53MKO (green bar) mice. (D) Plasma glucose levels of control and p53MKO mice after acute endurance running. (E) mRNA expression of *Slc2a4* in quadriceps and gastrocnemius of control and p53MKO mice after acute endurance exercise. Results presented as mean ± SEM and analysed by *t*‐test with Welch's correction (*n* = 4 in B, C; *n* = 9–14 in a, D, E); *P < 0.05, **P < 0.01, ***P < 0.001, ****P < 0.0001.

To verify whether the pre‐existing energy deficit induced by acute *Trp53*‐inactivation may impact on muscle performance, we next examined p53MKO mice during exercise. Reminiscent of the effects of food deprivation on gene expression, p53 target genes, *Cdkn1a*, *Ddit4*, and *Lpin1*, but not *Sesn2*, were induced in skeletal muscles following an acute endurance running bout in LOX‐control animals with intact *Trp53*, in which mice were prompted to run until exhaustion (Figure S11A). Comparing transcriptomic data from LOX‐control to p53MKO animals that were subjected to a treadmill run until exhaustion, KEGG pathway annotation also revealed changes to protein metabolism, circadian regulation, and FoxO signalling. Moreover, several nutrient‐related processes were downregulated in p53MKO after exercise, such as oxidative phosphorylation, calcium signalling, citrate cycle, and pyruvate metabolism (Tables S9 and S10). p53MKO mice showed reduced exercise capacity in comparison to controls, a reduced running distance and duration to exhaustion, and a trend towards lower running velocity at point of exhaustion (Figure 8A). Exercised p53MKO mice utilized more carbohydrates and less lipid as fuel compared with control animals, while VO2 and VCO2 were not significantly different (Figure S11B–H). These findings corresponded with a significantly earlier switch from lipids to carbohydrates as the primary energy source, known as the crossover point^22^ (Figure 8B,C). Additionally, post‐exercise p53MKO mice showed significantly lower plasma glucose levels (Figure 8D), which corresponded with increased expression of the glucose transporter 4 (GLUT4) gene, *Slc2a4*, in quadriceps and gastrocnemius muscles (Figure 8E). No significant differences were observed in plasma levels of other metabolites or insulin in p53MKO mice (Figure S12A, B). Paralleling the findings in p53MKO animals under *ad libitum* feeding and fasting, skeletal muscles from p53MKO mice after endurance exercise exhibited elevated expression of several fatty acid metabolism and transport genes (Figure S12C–E). Together, these findings suggest that p53MKO mice indeed utilize a higher proportion of carbohydrate as fuel, which may be achieved via increased glucose uptake from the circulation, although this remains to be investigated. Because p53MKO mice reach the crossover point to >50% carbohydrate fuel selection prematurely, an inability to acquire adequate glucose to sustain running at higher exercise intensity may lead to their earlier exhaustion compared with control animals.

## Discussion

The dynamics of acute metabolic adaptation to physiological challenges and nutrient availability in skeletal muscle remain incompletely understood. The results presented here highlight a previously unappreciated role of the p53 protein in maintaining energy homeostasis in myofibres, suggesting that p53 contributes to efficient nutrient/energy conservation under non‐stressed conditions. Acute *Trp53*‐deletion specifically in myofibres prompted a loss of catabolic repression, triggering futile macronutrient metabolization and generating a local energy deficit in skeletal muscle through glycogen depletion that mimics a fasting‐like state. As a tumour suppressor, p53 negatively regulates glycolysis, a process that is frequently augmented in cancer cells and consistent with the metabolic concepts of the Warburg hypothesis.^6^, ^23^ In our p53MKO model, skeletal muscles showed higher rates of carbohydrate oxidation and lactate formation and release, which led to severely diminished intramuscular glycogen reservoirs. Consistent with this observation, we previously demonstrated that acute deletion of p53 in liver reduced hepatic glycogen stores in mice.^4^, ^14^

Elevated locomotor activity is a characteristic response to food deprivation and is linked to foraging behaviour in multiple species.^21^, ^24^, ^25^ In mammals, it is additionally controlled by the central circadian clock.^26^ Diurnal rhythms of gene expression and metabolism within skeletal muscle are subject to control by the muscles' own circadian oscillations and *Trp53* has been implicated as a target of key circadian regulator, REV‐ERBα.^27^, ^28^ Hence, the shift in circadian gene expression patterns in the p53MKO model in the context of enhanced locomotion suggests that a muscle‐inherent, clock‐dependent mechanism may translate into increased activity. Previous reports have identified a direct association between p53 and core circadian regulators.^29^, ^30^ Intriguingly, this recent work uncovered shifts in metabolic gene expression following muscle‐specific ablation of BMAL1 that were similar to our p53MKO model, such as transient increases in the expression of genes regulating proteasomal degradation, autophagy and fatty acid oxidation. These findings provided evidence for a clock‐dependent regulation of muscle glucose metabolism and suggest that interactions between circadian rhythmicity regulators and p53 signalling may coordinate nutrient metabolism and the response to acute metabolic challenges, whereas an impaired muscle clock resulted in metabolic inefficiency.^28^

The depletion of muscle glycogen reservoirs in p53MKO mice likely reflects the premature switch of fuel utilization and reduced running capacity, as a pre‐existing suppression of glucose mobilization is absent. A key question to resolve in p53MKO mice is the fate of mobilized glucose. Firstly, increased systemic carbohydrate oxidation suggests that a proportion of glucose is metabolized fully by aerobic metabolism, potentially not in muscle itself, as PDH activity is reduced in muscles of p53MKO mice. Secondly, increased levels of the amino acid alanine in gastrocnemius suggest that transamination of glutamate and pyruvate to alanine and alpha‐ketoglutarate is elevated in p53MKO muscles. Muscle alanine is synthesized by the transamination of amino acids following proteolysis, a process that is upregulated during periods of energy deficit and is crucial for adaptation to fasting.^31^, ^32^ During this process, amino groups from the BCAAs leucine, isoleucine, and valine are transferred to alpha‐ketoglutarate to produce glutamate, which is subsequently deaminated by GPT2, simultaneously generating alanine from pyruvate. Alanine is transported to the liver for hepatic gluconeogenesis, forming one segment of the glucose‐alanine, or Cahill, cycle.^31^ BCAA‐derived alanine from skeletal muscle is rate‐limiting for hepatic gluconeogenesis in the response to fasting.^16^ Thirdly, we observed elevated circulating lactate levels in the initial knockout phase. Together with the inactivation of PDH, these observations suggest that in the early response to *Trp53* inactivation, the bulk of pyruvate from glycolysis is not converted to acetyl‐CoA, but rather to lactate for export. Therefore, the upregulation of the alanine and lactate pathways during the acute adaptation phase of genetic *Trp53* inactivation in skeletal muscle may also partially develop in response to the energy deficit caused by depleted glycogen reservoirs. Taken together, these findings indicate that the deletion of *Trp53* in myofibres promotes a shift towards catabolic processes that is comparable to fasted animals.

Unexpectedly, the effects of *Trp53* ablation in myofibres on muscle proteostasis were distinct in *ad libitum* fed and fasted conditions: while fed animals exhibited very mild signs of elevated autophagic markers, autophagy and proteasomal protein degradation markers were significantly blunted in p53MKO animals during fasting. Contrasting the fasting‐induced increase of BCAA in wild‐type muscles, intramuscular BCAA levels in fasted p53MKO animals were comparable to those found in *ad libitum* fed animals. A similar, although somewhat milder, effect is seen in regulation of carbohydrate metabolism, in which expression of some PDK isoforms is differentially regulated in p53MKO in the fed versus fasted states. These findings suggest that in *ad libitum* fed conditions, p53 deletion generates an energy deficient state locally in muscle that may promote proteolytic processes to provide additional fuel sources. In response to fasting, however, atrophy pathways that include the proteasomal‐ubiquitin and autophagy systems are compromised in mice with acute *Trp53* deletion. p53’s ability to regulate responses to metabolic stress is controlled by its subcellular localization. For instance, p53 can perform distinct functions in mitochondria, nuclei or the cytoplasm, and this may exert significant influence on the outcomes of p53 activation, which correlates with stressor intensity.^8^, ^33^, ^34^ Its localization to either mitochondria or nuclei induces differential effects of p53 on autophagy.^35^ Our previous work showed that fasting altered protein interaction partners of p53 depending on its sub‐cellular distribution, which included carbohydrate metabolism proteins, SORBS1 and UPG2.^36^ Such findings suggest that re‐distribution of p53 during fasting could explain some of the differential effects seen in p53MKO mice in fed and fasted states. p53‐dependent regulation of carbohydrate metabolism occurs in part through direct interaction with proteins such as PDK2, which suppresses *Pdk2* mRNA expression. However, kidney and colon tissue of p53‐deficient mice expressed lower baseline levels of *Pdk2*,^37^ indicating that other regulatory mechanisms might also be in place, such as FoxO and PPARs which could explain the distinctive transcriptional regulation in p53MKO in fed versus fasted states.

In summary, the findings reported in this study uncover a concept of energy substrate conservation under non‐stressed conditions that is acutely mediated by p53. Deletion of p53 in myofibres removes catabolic repression, promoting futile nutrient oxidation and disrupting the response to nutrient stress imposed by fasting and exercise. Because p53 signalling has also been implicated in obesity, a better mechanistic understanding of how p53 regulates metabolic flexibility and energy conservation in skeletal muscle could shed light on novel therapeutic targets in metabolic disease, cancer, and sarcopenia.

## Conflict of Interest

The authors declare no competing interests.

## Supporting information


**Figure S1.** mRNA expression of p53 target genes, *Cdkn1a, Ddit4, Lpin1*, and *Sesn2*, in skeletal muscles of the hindlimb and liver in mice fed *ad libitum* (white bars) and after 16‐hours fasting (grey bars). Results are presented as mean ± SEM and were analyzed by Mann‐Whitney test (n=8−10). * *p*<0.05, ** p<0.01.
**Figure S2.** **(A)** Schematic overview of the transgenic alleles and genotypes of p53MKO and LOX control mice, which were generated by intercrossing LOX mice, which carry loxP sequences (yellow arrow heads) flanking exons 2‐10 (E2‐10) of the *Trp53* gene, to mice expressing a tamoxifen‐inducible Cre‐recombinase / estrogen receptor fusion protein which was genetically modified to retain sensitivity to tamoxifen only (CreERT2). CreERT2 binds to the loxP sites upon tamoxifen administration and removes the DNA sequences between the two loxP sites, thereby generating the target gene (here: *Trp53*) inactivation. Expression of the CreERT2 protein is under control by the *Acta1*‐gene promoter, which results in myofiber‐specific expression of the recombinase. **(B)** Schematic overview of the experimental design used in this study: Mice were maintained on a phytoestrogen‐free diet before group assignment. Tamoxifen (i.p.‐injections: 2 mg/ 100 μL) was administered daily for five consecutive days and animals were allowed a short period of 48 hours for recovery before being killed for tissue collection with and without a 16‐hour fast (termed “standard experiment”). In a second experimental setup (termed: “short‐term tamoxifen and 4h‐fasting”, animals were killed on the evening of the third tamoxifen administration with and with a short, 4‐hour fast. **(C)** mRNA expression of Trp53 in skeletal muscles of the hindlimb from control (white bars) and p53MKO (blue bars) mice (n=13‐14). **(D)** Body mass, **(E)** fat and lean mass assessed by NMR body composition analysis normalized to total body weight, **(F)** skeletal muscle masses normalized to body weight (n=13‐14), and **(G)** gene transcript levels (RNAseq) of *Myh2, Myh1, Myh7*, and *Myh4* in quadriceps muscle of control (with bars) and p53MKO (blue bars) mice (n=3‐5). **(H)** ImageJ‐based analysis of myofiber cross‐sectional areas (CSA; left panels) and quantifications (right panel) of average CSAs in fed control (white bar), fed p53MKO (light blue bar), fasted control (grey bar) and fasted p53MKO (dark blue bar) mice n=3). Results are presented as mean ± SEM and were analyzed by Mann‐Whitney test (n=8‐10). * *p*<0.05, ** *p*<0.01, *** *p*<0.001, **** *p*<0.0001.
**Figure S3.** **(A)** Respiratory exchange ratio (RER), **(B)** carbohydrate (CH) oxidation rate, **(C)** lipid oxidation rate, **(D)** average light and dark phase RER, **(E)** average light and dark phase carbohydrate oxidation rate, and **(F)** average lipid oxidation rate in control (black line; white bars) and p53MKO (blue line; blue bars) mice in light (white background) and dark (grey background) phases. **(G)** Plasma corticosterone and **(H)** cortisol in control (white bars) and p53MKO (blue bars) mice. **(I)** Respiratory exchange ratio (RER), **(J)** oxygen consumption (VO2), **(K)** energy expenditure (EE), **(L)** carbohydrate oxidation, and **(M)** lipid oxidation, measured by indirect calorimetry in control and p53MKO under fed conditions (control: black line; p53MKO: blue line) and in response to fasting (control: grey line; p53MKO: red line; starting time of food withdrawal at 4 pm is indicated by broken line). Results presented as mean ± SEM and compared by Mann Whitney test, 2‐way ANOVA, or multiple t‐tests. *n* = 4‐5 (A‐F); *n* = 5‐7 (G, H), *n* = 6‐12 (all other panels). * *p*<0.05, ** *p*<0.01 (fed: control *vs*. p53MKO); # *p*<0.05 (fasted: control *vs*. p53MKO).
**Figure S4.** Heatmap of differentially expressed genes (*p*<0.05; Z‐scores) related to **(A)** proteolysis and **(B)** protein synthesis from quadriceps muscle of control and p53MKO animals under fed and fasted conditions. Results expressed as Z‐score (*n*=3‐5).
**Figure S5.** **(A)** Amino acids, lysine, glycine, aspartate, isoleucine, leucine, and valine, and alanine in gastrocnemius muscle under fed (control: white bars; p53MKO: light blue bars) and fasted (control: grey bars; p53MKO: dark blue bars) conditions, measured using NMR spectroscopy (n=3‐5). **(B)** mRNA expression of Gpt2 in quadriceps muscle (n=8‐10). **(C)** Expression of autophagy‐related gene transcripts from RNAseq analysis (expressed as normalized gene counts; log2 of quadriceps muscle; n=3‐5). **(D)** Protein level of K48‐linked protein ubiquitination in quadriceps, with quantification (right panel) normalized to total protein (n=5‐10). **(E)** Protein level of K63‐linked protein ubiquitination in quadriceps, with quantification (right panel) normalized to total protein (n=4‐8). **(F)** 26S‐Proteasome activity in quadriceps muscle (n=4‐5). Results presented as mean ± SEM and compared by 2‐way ANOVA or Mann‐Whitney test; * *p*<0.05, ** *p*<0.01, *** *p*<0.001, **** *p*<0.0001.
**Figure S6.** **(A)** Levels of glycogen, glucose, pyruvate, and lactate in gastrocnemius muscle under fed (control: white bars; p53MKO: blue bars) and fasted (control: grey bars; p53MKO: navy bars) conditions, measured by NMR spectroscopy (n=3‐5). **(B)** PDH activity in gastrocnemius muscle (n=3‐5). **(C)** mRNA expression of *Pdk2, Pdk4, Pdp1*, and *Pdha1* in quadriceps and **(D)** gastrocnemius muscle of fed control and p53MKO mice (n=7‐9). **(E)** Citrate synthase activity in gastrocnemius muscle (n=4). **(F, G)** Representative immunodetections (F) and quantifications (G) of complexes I‐V of the mitochondrial respiratory chain of oxidative phosphorylation in quadriceps muscle under fed and fasted conditions and normalized to total PDH and total protein loading (n=4). **(H)** (D) Gene expression of genes regulating fatty acid oxidation in quadriceps muscle from control and p53MKO mice conditions (n=6‐11). Results presented as mean ± SEM and compared by 2‐way ANOVA or Mann‐Whitney test; * *p*<0.05, ** *p*<0.01, *** *p*<0.001, **** *p*<0.0001.
**Figure S7.** **(A)** Lipid oxidation, **(B)** energy expenditure, **(C)** food intake in control (black line) and p53MKO (blue line) mice during and immediately after tamoxifen administration (n=3‐5). Red arrows indicate days of tamoxifen administration. **(D)** Cumulative food intake comparing LOX‐control (white bars) to p53MKO (blue bars) throughout the full time‐course, during the tamoxifen‐administration phase, and during the 48 hours of recovery after the last tamoxifen administration. Results presented as mean ± SEM and compared by multiple t‐tests; * *p*<0.05.
**Figure S8.** **(A)** Gene expression of gluconeogenic genes *Gpt1, Pcx, Pck1*, and **G6pc** in liver of fed (control: white bars; p53MKO: light blue bars) and fasted (control: grey bars; p53MKO: dark blue bars) mice (n=3‐5). Hepatic **(B)** triglyceride and **(C)** glycogen levels (n=3‐5). **(D)** Inguinal white adipose tissue (WAT) and **(E)** epididymal WAT mass (n=3‐5). **(F)** Representative immunodetections (left panels) and quantifications (right panels) of phosphorylated PDH (p‐PDH at S293), phosphorylated PDH (p‐PDH at S300), pyruvate carboxylase (PCX) in quadriceps muscle from control and p53MKO mice during fed conditions (control: white bars; p53MKO: yellow bars) and following 4‐hours of food withdrawal (4 pm to 8 pm; control: grey bars; p53MKO: orange bars) in mice directly after an abbreviated course of short‐term (3‐day) tamoxifen administration. Quantifications were normalized to total corresponding protein (for p‐PDH normalized to total PDH (t‐PDH) depicted in main figure 7D) and total protein (TP) loading (n=3‐5). Results presented as mean ± SEM and compared by 2‐way ANOVA, Mann‐Whitney test, or multiple t‐tests; * p<0.05, ** p<0.01, *** p<0.001, *** p<0.001.
**Figure S9.** **(A)** Representative detection (left panel) of protein levels of LC3‐II and LC3‐I in quadriceps muscle of short‐term (3‐day) tamoxifen treated control and p53MKO mice in the fed state (control: white bars; p53MKO: blue bars) and following 4‐hours of food withdrawal (control: grey bars; p53MKO: dark blue bars) and quantification of LC3II:LC3I ratio normalized to total protein (right panel). **(B)** mRNA expression of *Fbxo32* and *Trim63* in quadriceps muscle under similar conditions. Results presented as mean ± SEM and compared by 2‐way ANOVA (n=4‐5 for A and B); * p<0.05, ** p<0.01, *** p<0.001, **** p<0.0001.
**Figure S10.** **(A)** Circulating free fatty acids (FFA), triglycerides (TG), urea, and (B) insulin in control and p53MKO mice in the fed state (control: white bars; p53MKO: blue bars) and following 16‐hours of food withdrawal (control: grey bars; p53MKO: dark blue bars) in mice the received the full course of 5‐day tamoxifen treatment (n=10‐11). **(C)** Circulating free fatty acids (FFA) and triglycerides (TG) in control and p53MKO mice in the fed state (control: white bars; p53MKO: yellow bars) and following 4‐hours of food withdrawal (control: grey bars; p53MKO: orange bars) in mice the received the full course of 5‐day tamoxifen treatment (n=7‐8); * p<0.05, ** p<0.01, *** p<0.001, **** p<0.0001.
**Figure S11.** **(A)** Gene expression of p53 target genes *Cdkn1a, Ddit4, Lpin1*, and *Sesn2* in wild‐type C57BL/6J mice at rest (white bars) and after an acute endurance treadmill run until exhaustion (grey bars). Control (black line) and p53MKO (green line) mice underwent an acute endurance treadmill run in a metabolic chamber, in which gas exchange was measured (n=3‐5). **(B)** Carbohydrate oxidation rate, **(C)** percent carbohydrate oxidation, **(D)** lipid oxidation rate, **(E)**percent lipid oxidation, **(F)** respiratory exchange ratio (RER), **(G)** oxygen consumption (VO2), **(H)** carbon dioxide expiration (VCO2) in control and p53MKO animals (n=4). The dashed line in panel F represents the point at which fuel utilization is equal between carbohydrate and lipid substrates. Results presented as mean ± SEM and compared by Mann‐Whitney test or multiple t‐tests; * p<0.05, ** p<0.01, *** p<0.001.
**Figure S12.** **(A)** Plasma metabolites and **(B)** insulin hormone in control (white bars) and p53MKO (green bars) animals after acute endurance run. Gene expression of genes regulating fatty acid oxidation in **(C)** quadriceps and **(D)** gastrocnemius muscle from control and p53MKO mice. **(E)** Normalized gene counts (log2) of fatty acid metabolism genes from RNAseq analysis of quadriceps muscle. Results presented as mean ± SEM and compared by Mann‐Whitney test (n=3‐4 in all panels).

**Table S1.** Transcriptomic analysis of upregulated KEGG pathways in quadriceps muscle by fasting (16 hours) compared with fed mice.
**Table S2.** Transcriptomic analysis of downregulated KEGG pathways in quadriceps muscle by fasting (16 hours) compared with fed mice.
**Table S3.** Transcriptomic analysis of upregulated genes (upregulated by fasting [16 hours] compared with fed control mice) appearing in KEGG pathway analysis of quadriceps muscle.
**Table S4.** Transcriptomic analysis of downregulated genes (downregulated by fasting [16 hours] compared with fed control mice) appearing in KEGG pathway analysis of quadriceps muscle.
**Table S5.** Transcriptomic analysis of upregulated KEGG pathways in quadriceps muscle of p53MKO mice compared to control mice with ad libitum feeding.
**Table S6.** Transcriptomic analysis of downregulated KEGG pathways in quadriceps muscle of p53MKO mice compared to control mice with ad libitum feeding.
**Table S7.** Transcriptomic analysis of upregulated gene ontology terms (biological processes) in quadriceps muscle of p53MKO mice compared to control mice with ad libitum feeding.
**Table S8.** Transcriptomic analysis of downregulated gene ontology terms (biological processes) in quadriceps muscle of p53MKO mice compared to control mice with ad libitum feeding.
**Table S9.** Transcriptomic analysis of upregulated KEGG pathways in quadriceps muscle of p53MKO mice compared to control mice, after acute endurance exercise.
**Table S10.** Transcriptomic analysis of downregulated KEGG pathways in quadriceps muscle of p53MKO mice compared to control mice, after acute endurance exercise.

**Table S11.** Primer sequences for qPCR analyses.
