# Supplementary material for: Skeletal muscle p53‐depletion uncovers a mechanism of fuel usage suppression that enables efficient energy conservation
Source: J Cachexia Sarcopenia Muscle. 2024 Jul 15;15(5):1772–84. doi: 10.1002/jcsm.13529 (PMC11446685; doi:10.1002/jcsm.13529)
Supplement: Supplementary file 1 — Figure S1. mRNA expression of p53 target genes, Cdkn1a, Ddit4, Lpin1, and Sesn2, in skeletal muscles of the hindlimb and liver in mice fed ad libitum (white bars) and after 16‐hours fasting (grey bars). Results are presented as mean ± SEM and were analyzed by Mann‐Whitney test (n=8−10). * p<0.05, ** p<0.01. Figure S2. (A) Schematic overview of the transgenic alleles and genotypes of p53MKO and LOX control mice, which were generated by intercrossing LOX mice, which carry loxP sequences (yellow arrow heads) flanking exons 2‐10 (E2‐10) of the Trp53 gene, to mice expressing a tamoxifen‐inducible Cre‐recombinase / estrogen receptor fusion protein which was genetically modified to retain sensitivity to tamoxifen only (CreERT2). CreERT2 binds to the loxP sites upon tamoxifen administration and removes the DNA sequences between the two loxP sites, thereby generating the target gene (here: Trp53) inactivation. Expression of the CreERT2 protein is under control by the Acta1‐gene promoter, which results in myofiber‐specific expression of the recombinase. (B) Schematic overview of the experimental design used in this study: Mice were maintained on a phytoestrogen‐free diet before group assignment. Tamoxifen (i.p.‐injections: 2 mg/ 100 μL) was administered daily for five consecutive days and animals were allowed a short period of 48 hours for recovery before being killed for tissue collection with and without a 16‐hour fast (termed “standard experiment”). In a second experimental setup (termed: “short‐term tamoxifen and 4h‐fasting”, animals were killed on the evening of the third tamoxifen administration with and with a short, 4‐hour fast. (C) mRNA expression of Trp53 in skeletal muscles of the hindlimb from control (white bars) and p53MKO (blue bars) mice (n=13‐14). (D) Body mass, (E) fat and lean mass assessed by NMR body composition analysis normalized to total body weight, (F) skeletal muscle masses normalized to body weight (n=13‐14), and (G) gene transcript levels (RNAseq) [file JCSM-15-1772-s001.pdf]

Supplementary information

Supplementary figures

Supplementary figure and table legends

Methods

Supplementary Figure S1

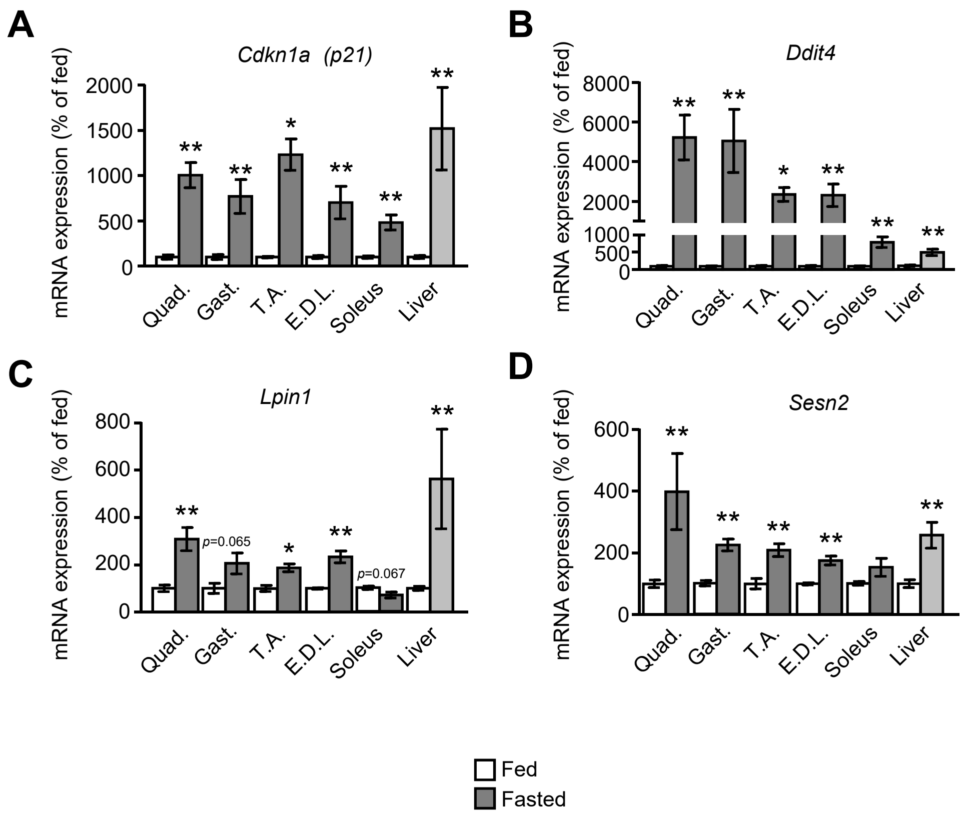

Supplementary Figure S2

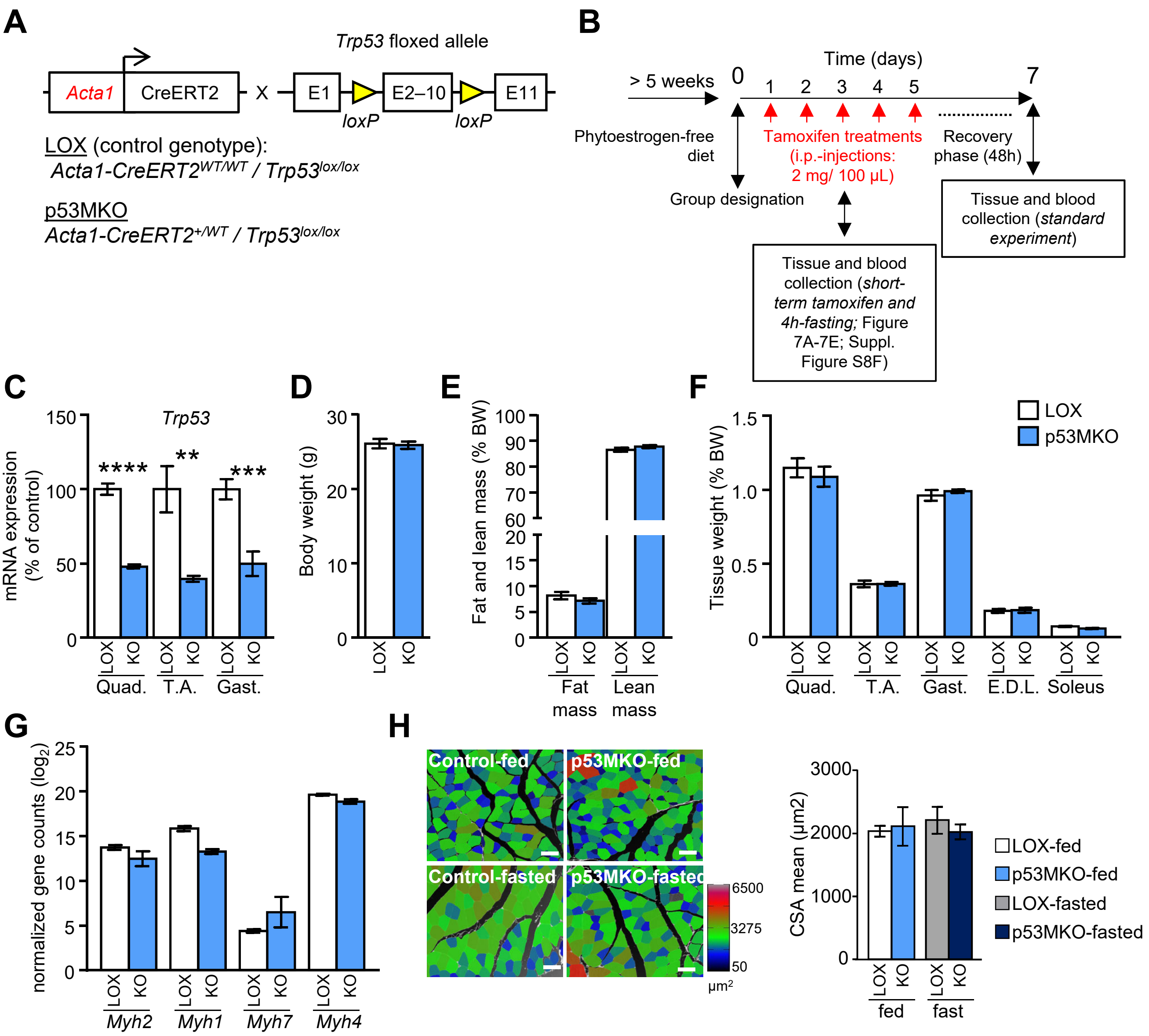

Supplementary Figure S3

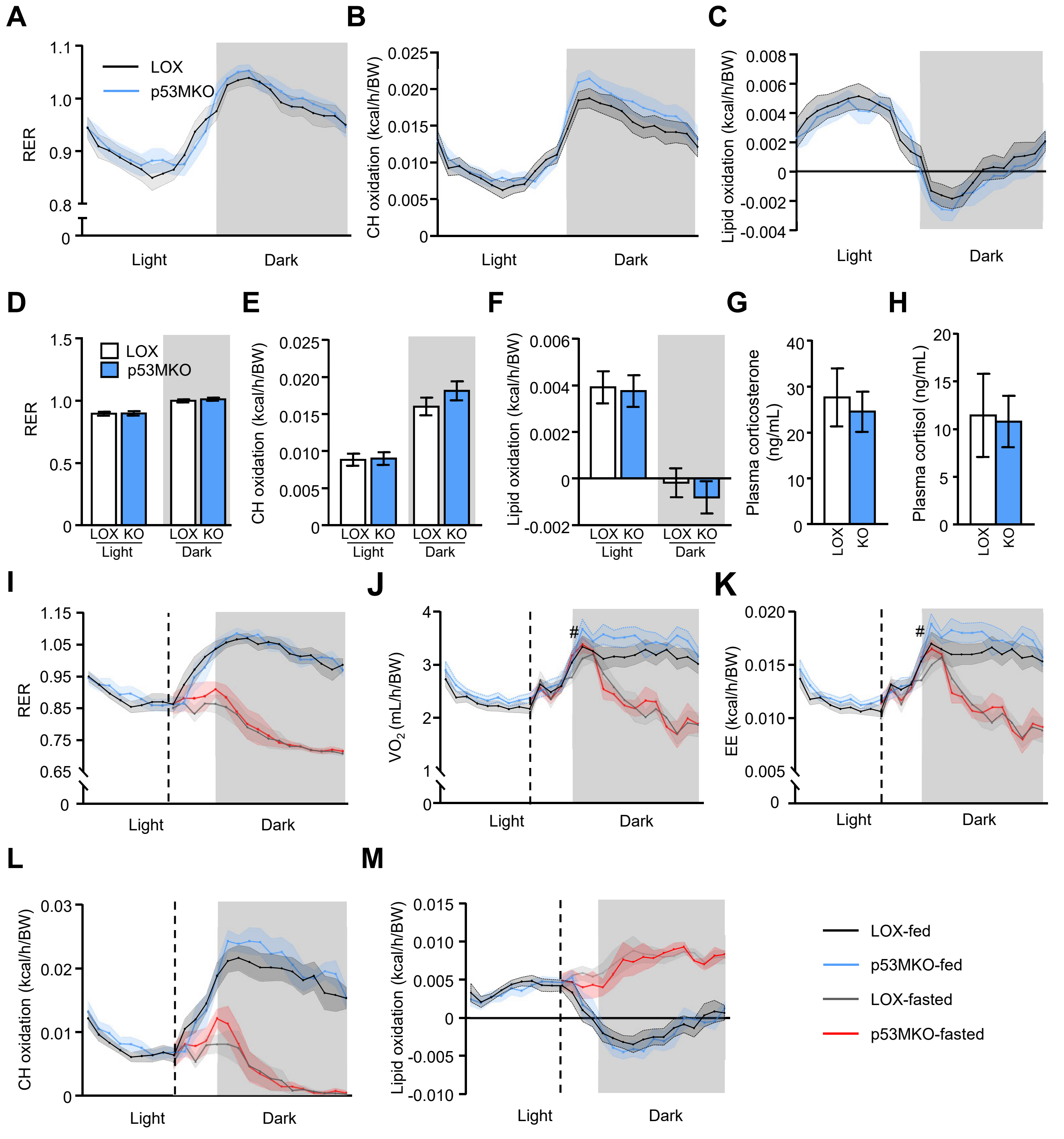

Supplementary Figure S4

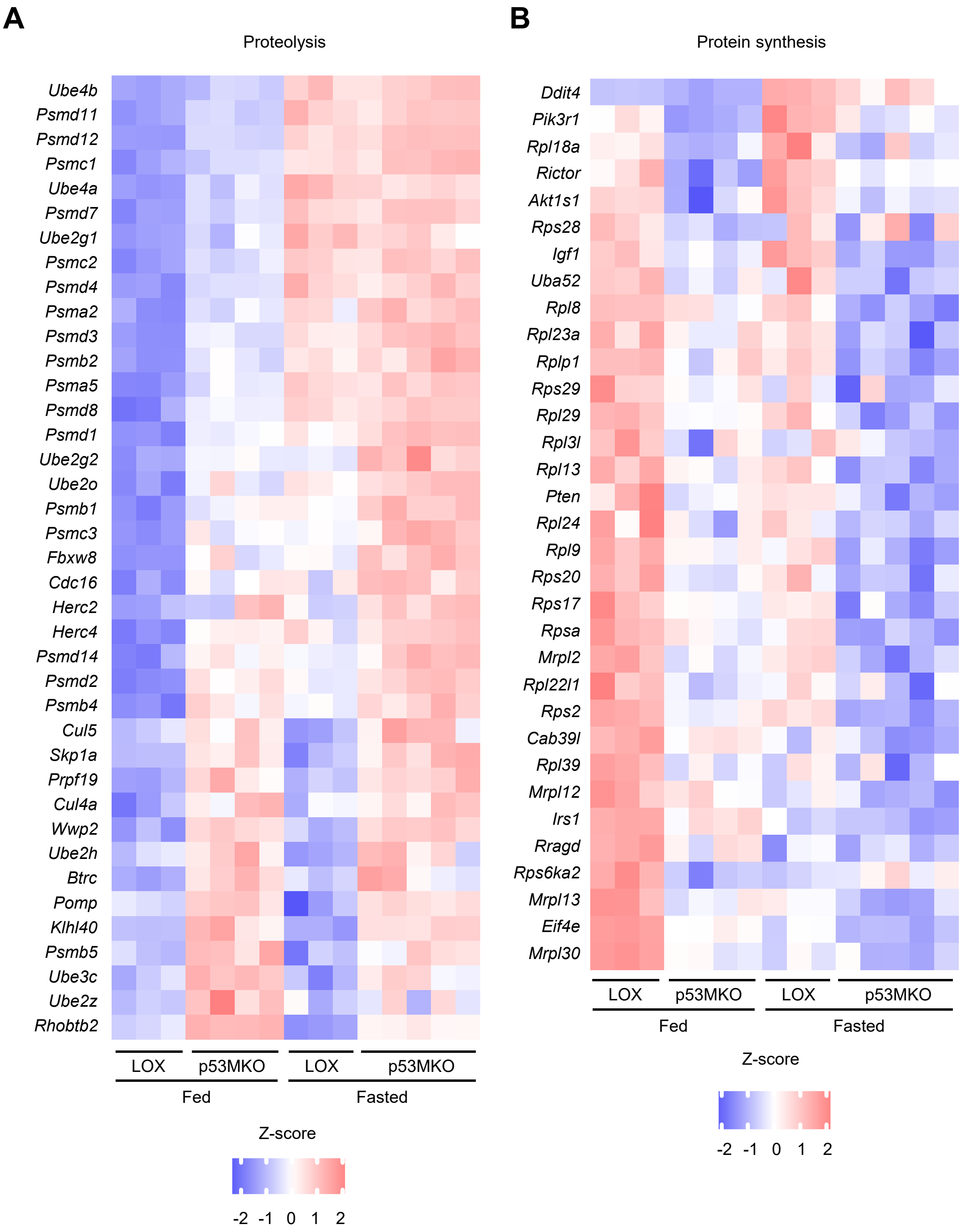

Supplementary Figure S5

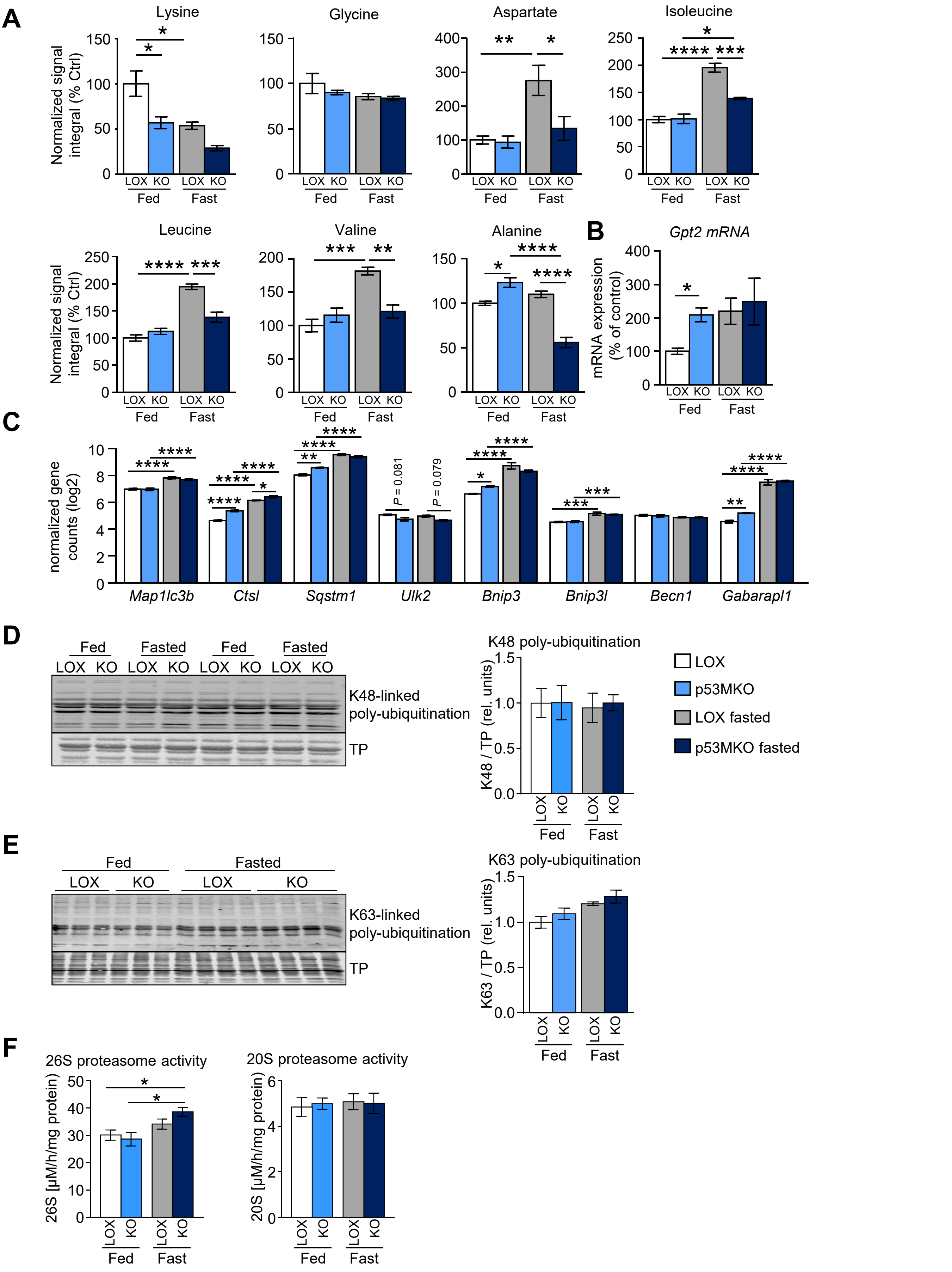

Supplementary Figure S6

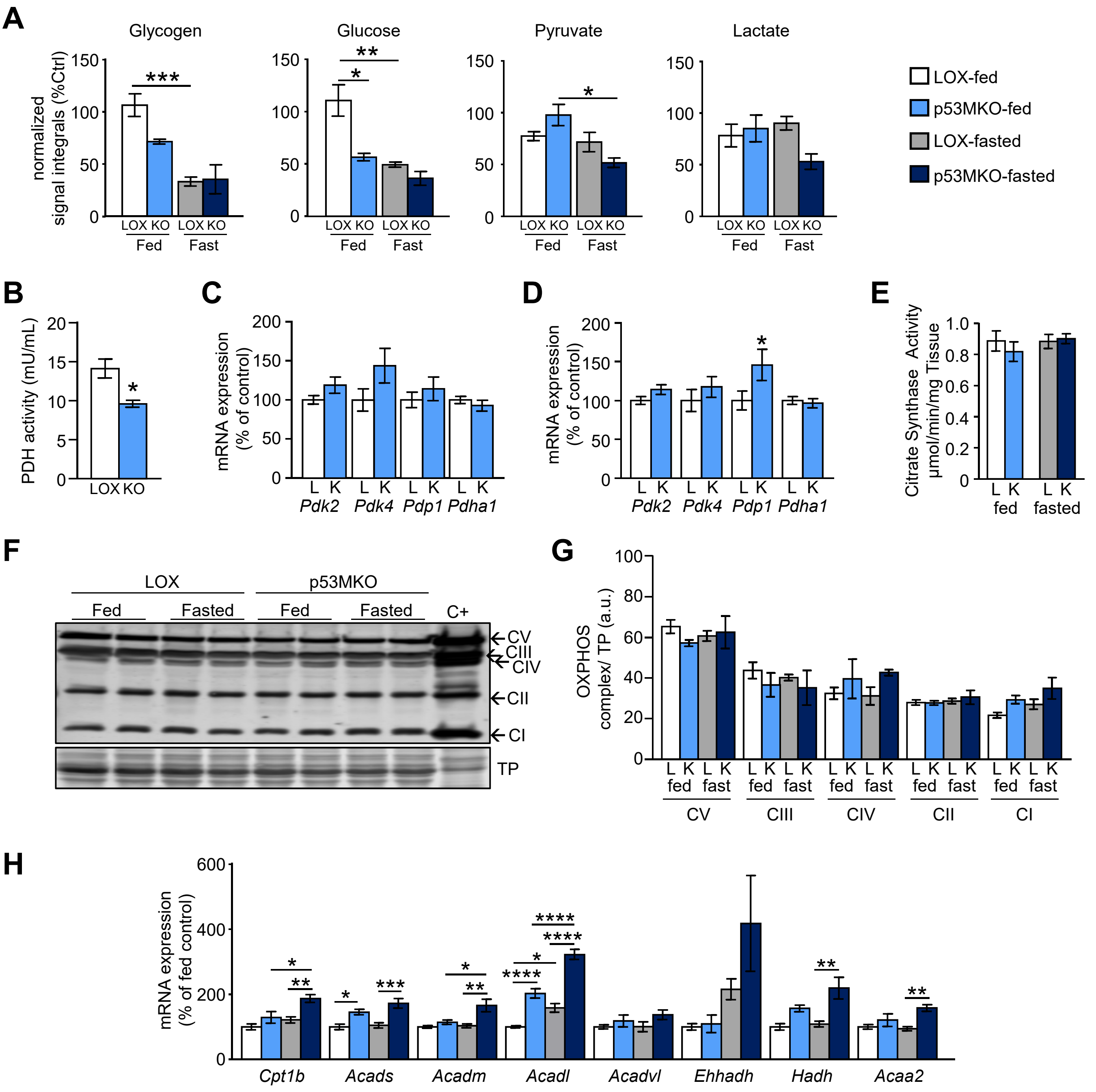

Supplementary Figure S7

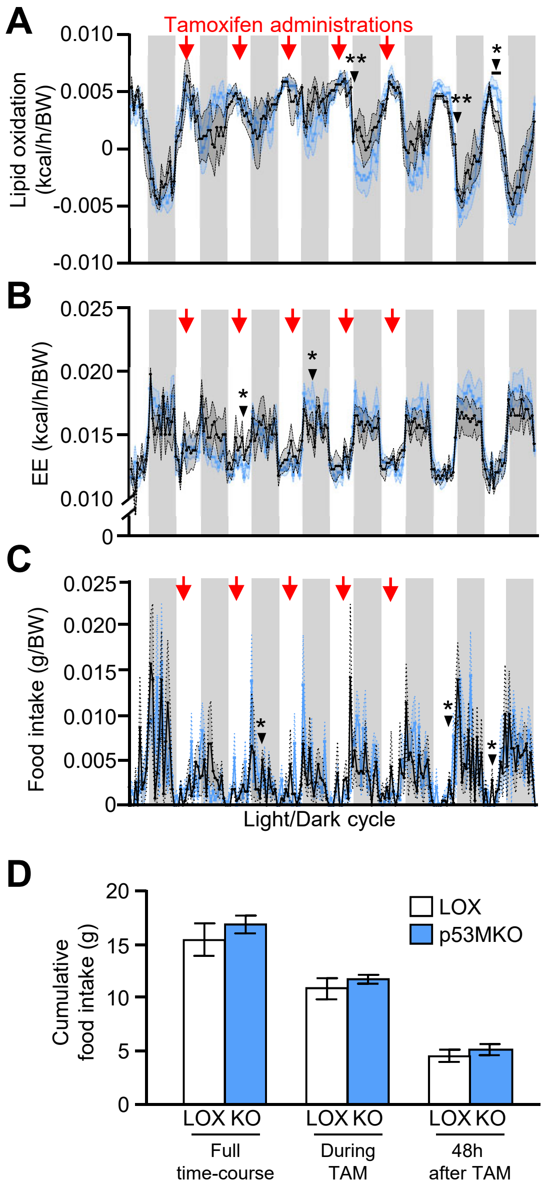

Supplementary Figure S8

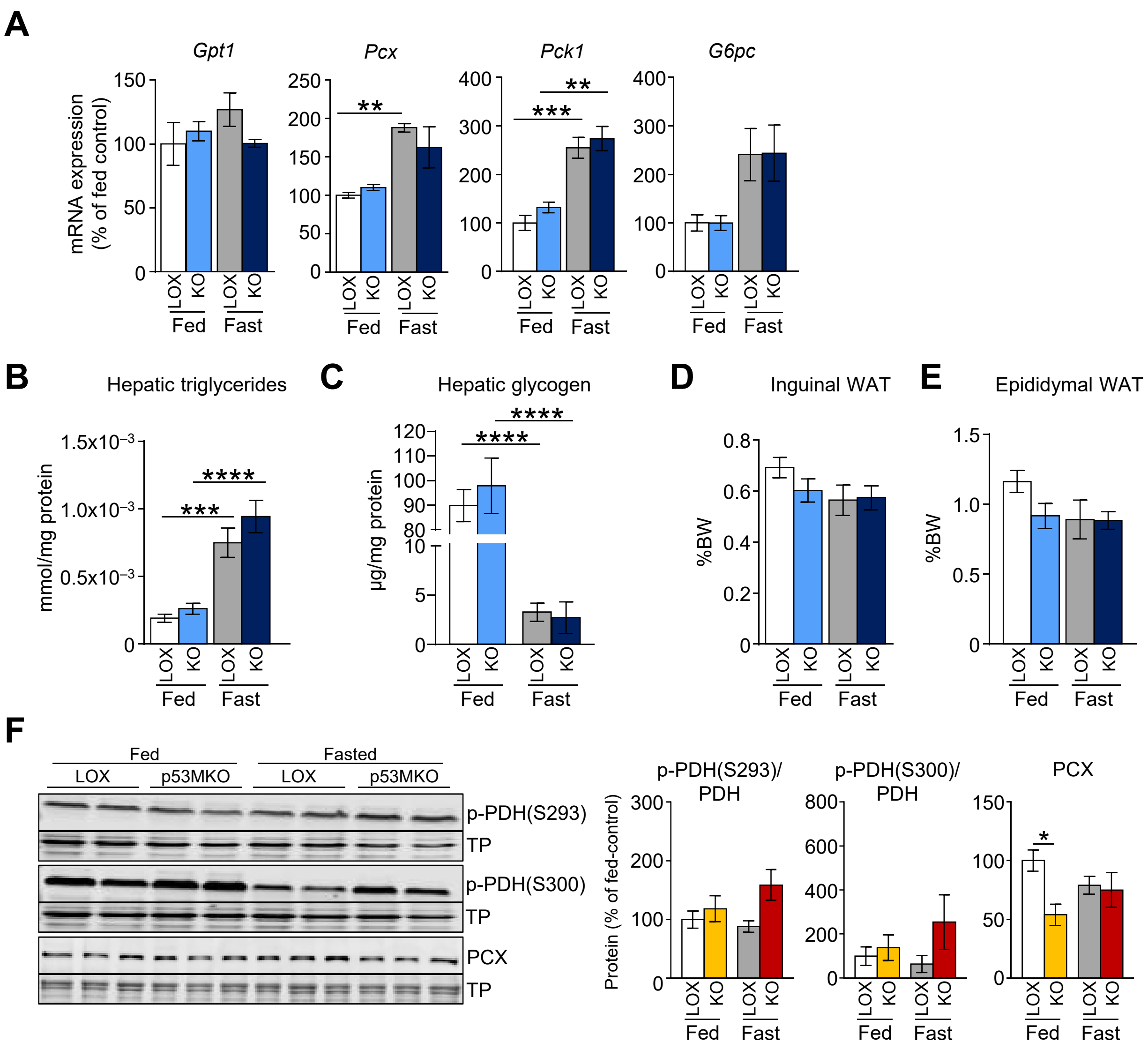

Supplementary Figure S9

A

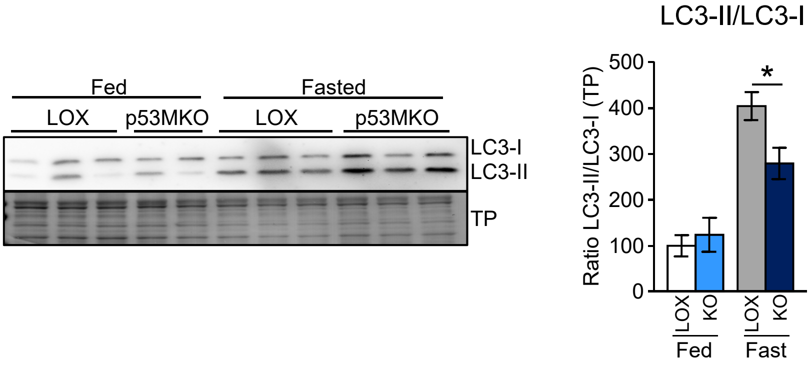

B

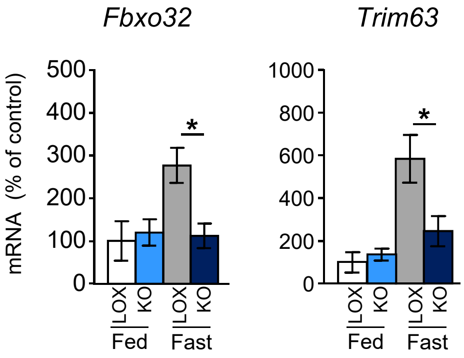

Supplementary Figure S10

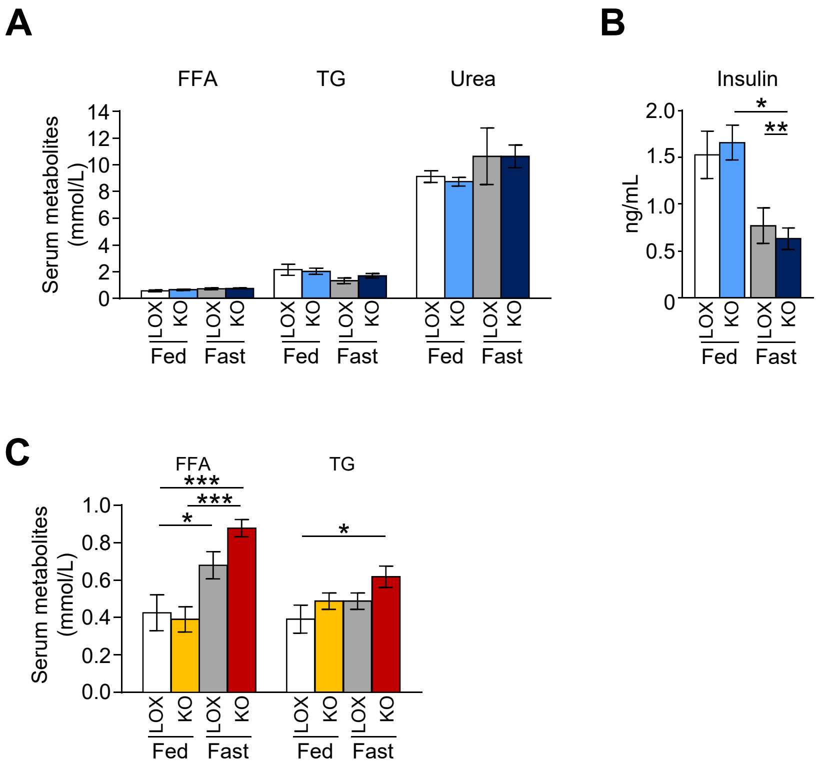

Supplementary Figure S11

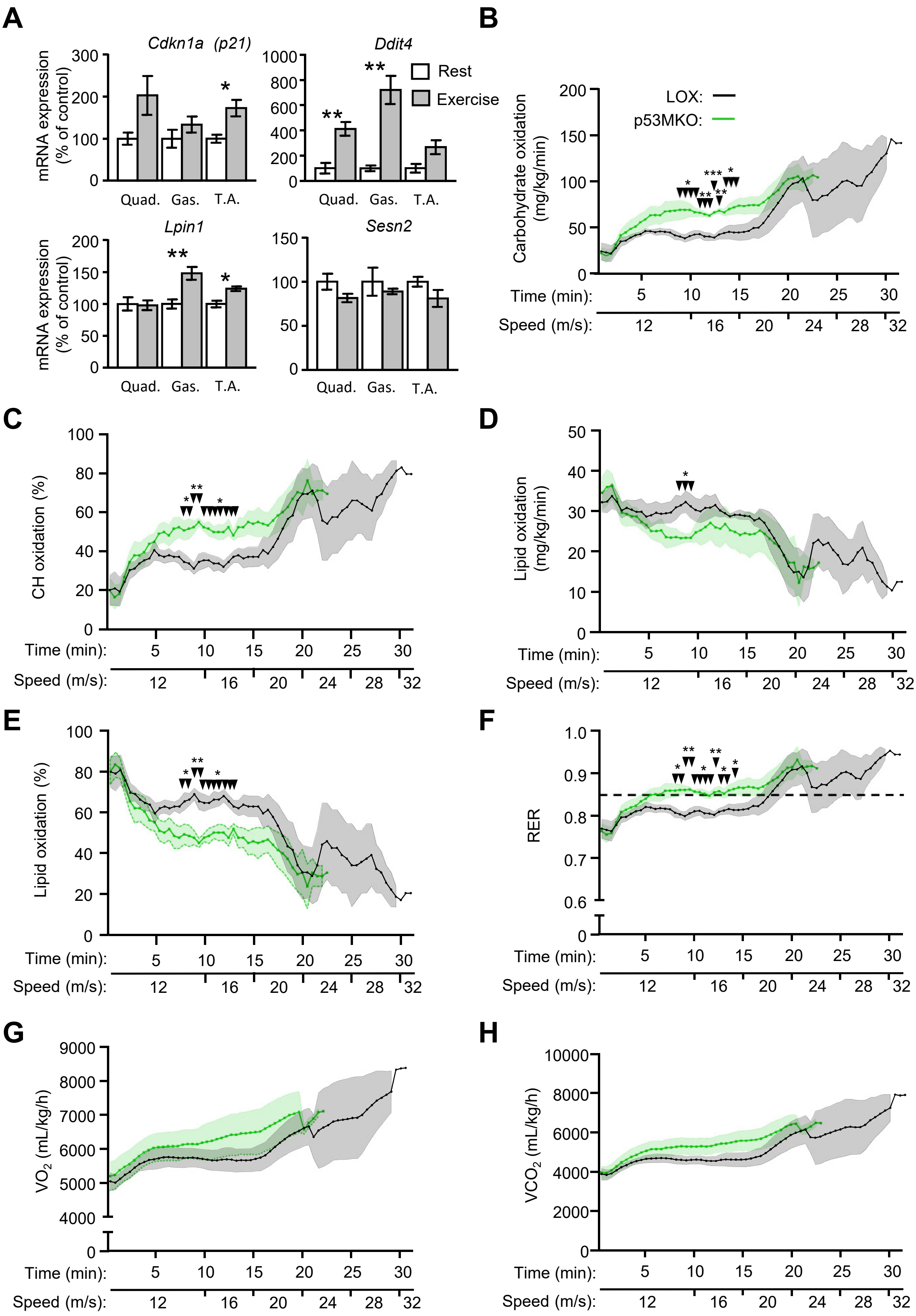

Supplementary Figure S12

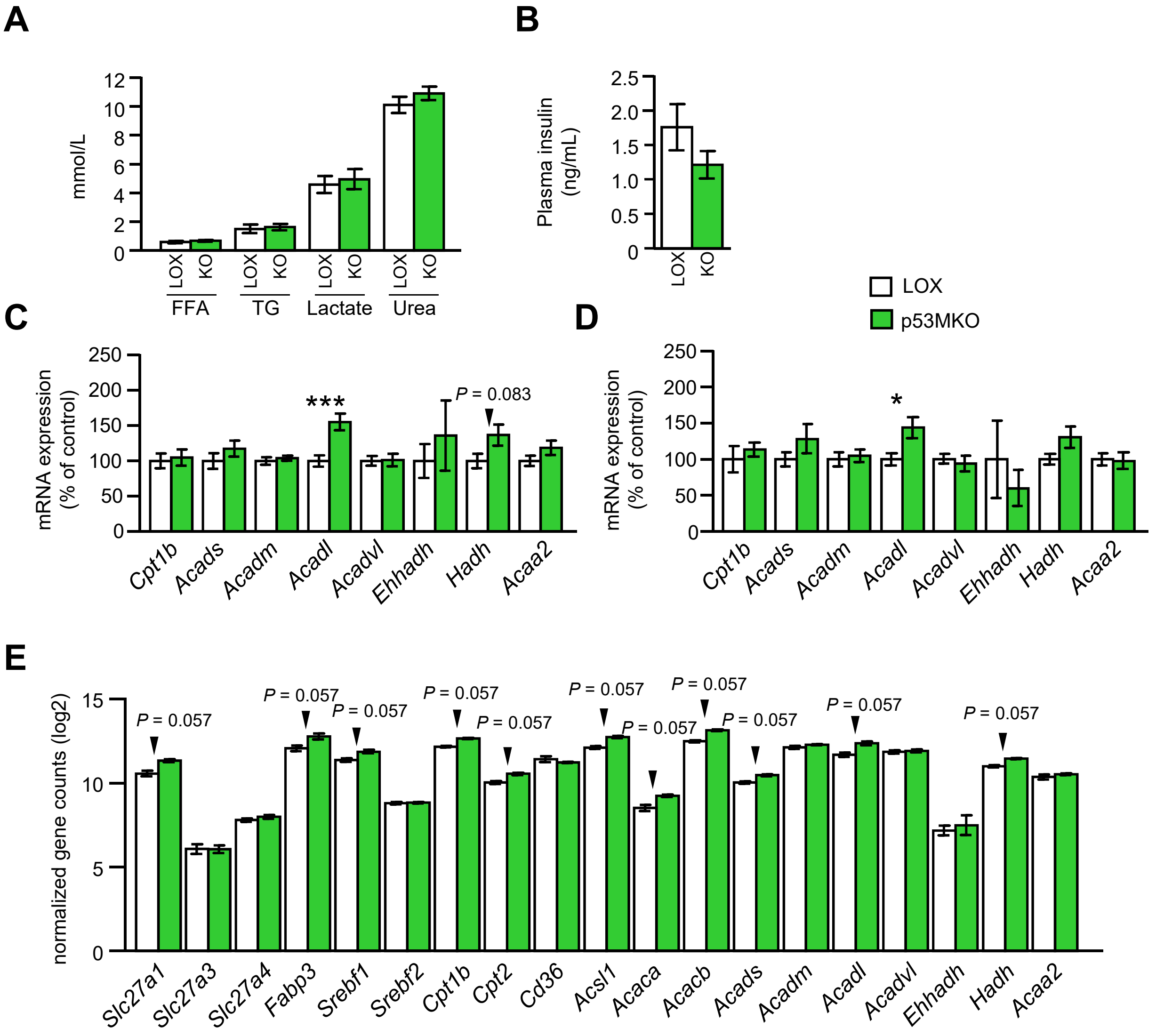

Supplementary Figure legends

Figure S1. mRNA expression of p53 target genes, *Cdkn1a*, *Ddit4*, *Lpin1*, and *Sesn2,* in skeletal muscles of the hindlimb and liver in mice fed *ad libitum* (white bars) and after 16-hours fasting (grey bars). Results are presented as mean ± SEM and were analyzed by Mann-Whitney test (n=8–10). * $p<0.05$, ** $p<0.01$.

Figure S2. (**A**) Schematic overview of the transgenic alleles and genotypes of p53MKO and LOX control mice, which were generated by intercrossing LOX mice, which carry loxP sequences (yellow arrow heads) flanking exons 2-10 (E2-10) of the *Trp53* gene, to mice expressing a tamoxifen-inducible Cre-recombinase / estrogen receptor fusion protein which was genetically modified to retain sensitivity to tamoxifen only (CreERT2). CreERT2 binds to the loxP sites upon tamoxifen administration and removes the DNA sequences between the two loxP sites, thereby generating the target gene (here: *Trp53*) inactivation. Expression of the CreERT2 protein is under control by the *Acta1*-gene promoter, which results in myofiber-specific expression of the recombinase. (**B**) Schematic overview of the experimental design used in this study: Mice were maintained on a phytoestrogen-free diet before group assignment. Tamoxifen (i.p.-injections: 2 mg/ 100 µL) was administered daily for five consecutive days and animals were allowed a short period of 48 hours for recovery before being killed for tissue collection with and without a 16-hour fast (termed “standard experiment”). In a second experimental setup (termed: “short-term tamoxifen and 4h-fasting”, animals were killed on the evening of the third tamoxifen administration with and with a short, 4-hour fast. (**C**) mRNA expression of *Trp53* in skeletal muscles of the hindlimb from control (white bars) and p53MKO (blue bars) mice (n=13-14). (**D**) Body mass, (**E**) fat and lean mass assessed by NMR body composition analysis normalized to total body weight, (**F**) skeletal muscle masses normalized to body weight (n=13-14), and (**G**) gene transcript levels (RNAseq) of *Myh2*, *Myh1*, *Myh7*, and *Myh4* in quadriceps muscle of control (with bars) and p53MKO (blue bars) mice (n=3-5). (**H**) ImageJ-based analysis of myofiber cross-sectional areas (CSA; left panels) and quantifications (right panel) of average CSAs in fed control (white bar), fed p53MKO (light blue bar), fasted control (grey bar) and fasted p53MKO (dark blue bar) mice n=3). Results are presented as mean ± SEM and were analyzed by Mann-Whitney test (n=8–10). * $p<0.05$, ** $p<0.01$, *** $p<0.001$, **** $p<0.0001$.

Figure S3. (**A**) Respiratory exchange ratio (RER), (**B**) carbohydrate (CH) oxidation rate, (**C**) lipid oxidation rate, (**D**) average light and dark phase RER, (**E**) average light and dark phase carbohydrate oxidation rate, and (**F**) average lipid oxidation rate in control (black line; white bars) and p53MKO (blue line; blue bars) mice in light (white background) and dark (grey background) phases. (**G**) Plasma corticosterone and (**H**) cortisol in control (white bars) and p53MKO (blue bars) mice. (**I**) Respiratory exchange ratio (RER), (**J**) oxygen consumption (VO_2), (**K**) energy expenditure (EE), (**L**) carbohydrate oxidation, and (**M**) lipid oxidation, measured by indirect calorimetry in control and p53MKO under fed conditions (control: black line; p53MKO: blue line) and in response to fasting (control: grey line; p53MKO: red line; starting time of food withdrawal at 4 pm is indicated by broken line). Results presented as mean ± SEM and compared by Mann Whitney test, 2-way ANOVA, or multiple t-tests. *n* = 4–5 (A–F); *n* = 5–7 (G, H), *n* = 6–12 (all other panels). * p<0.05, ** p<0.01 (fed: control *vs*. p53MKO); # p<0.05 (fasted: control *vs*. p53MKO).

Figure S4. Heatmap of differentially expressed genes (p<0.05; Z-scores) related to (**A**) proteolysis and (**B**) protein synthesis from quadriceps muscle of control and p53MKO animals under fed and fasted conditions. Results expressed as Z-score (*n*=3–5).

Figure S5. (**A**) Amino acids, lysine, glycine, aspartate, isoleucine, leucine, and valine, and alanine in gastrocnemius muscle under fed (control: white bars; p53MKO: light blue bars) and fasted (control: grey bars; p53MKO: dark blue bars) conditions, measured using NMR spectroscopy (n=3-5). (**B**) mRNA expression of Gpt2 in quadriceps muscle (n=8-10). (**C**) Expression of autophagy-related gene transcripts from RNAseq analysis (expressed as normalized gene counts; log2 of quadriceps muscle; n=3-5). (**D**) Protein level of K48-linked protein ubiquitination in quadriceps, with quantification (right panel) normalized to total protein (n=5-10). (**E**) Protein level of K63-linked protein ubiquitination in quadriceps, with quantification (right panel) normalized to total protein (n=4-8). (**F**) 26S-Proteasome activity in quadriceps muscle (n=4-5). Results presented as mean ± SEM and compared by 2-way ANOVA or Mann-Whitney test; * p<0.05, ** p<0.01, *** p<0.001, **** p<0.0001.

Figure S6. (**A**) Levels of glycogen, glucose, pyruvate, and lactate in gastrocnemius muscle under fed (control: white bars; p53MKO: blue bars) and fasted (control: grey bars; p53MKO: navy bars) conditions,

measured by NMR spectroscopy (n=3-5). (**B**) PDH activity in gastrocnemius muscle (n=3-5). (**C**) mRNA expression of *Pdk2*, *Pdk4*, *Pdp1*, and *Pdha1* in quadriceps and (**D**) gastrocnemius muscle of fed control and p53MKO mice (n=7-9). (**E**) Citrate synthase activity in gastrocnemius muscle (n=4). (**F, G**) Representative immunodetections (F) and quantifications (G) of complexes I-V of the mitochondrial respiratory chain of oxidative phosphorylation in quadriceps muscle under fed and fasted conditions and normalized to total PDH and total protein loading (n=4). (**H**) (D) Gene expression of genes regulating fatty acid oxidation in quadriceps muscle from control and p53MKO mice conditions (n=6-11). Results presented as mean ± SEM and compared by 2-way ANOVA or Mann-Whitney test; * $p<0.05$, ** $p<0.01$, *** $p<0.001$, **** $p<0.0001$.

Figure S7. (**A**) Lipid oxidation, (**B**) energy expenditure, (**C**) food intake in control (black line) and p53MKO (blue line) mice during and immediately after tamoxifen administration (n=3-5). Red arrows indicate days of tamoxifen administration. (**D**) Cumulative food intake comparing LOX-control (white bars) to p53MKO (blue bars) throughout the full time-course, during the tamoxifen-administration phase, and during the 48 hours of recovery after the last tamoxifen administration. Results presented as mean ± SEM and compared by multiple t-tests; * $p<0.05$.

Figure S8. (**A**) Gene expression of gluconeogenic genes *Gpt1*, *Pcx*, *Pck1*, and *G6pc* in liver of fed (control: white bars; p53MKO: light blue bars) and fasted (control: grey bars; p53MKO: dark blue bars) mice (n=3-5). Hepatic (**B**) triglyceride and (**C**) glycogen levels (n=3-5). (**D**) Inguinal white adipose tissue (WAT) and (**E**) epididymal WAT mass (n=3-5). (**F**) Representative immunodetections (left panels) and quantifications (right panels) of phosphorylated PDH (p-PDH at S293), phosphorylated PDH (p-PDH at S300), pyruvate carboxylase (PCX) in quadriceps muscle from control and p53MKO mice during fed conditions (control: white bars; p53MKO: yellow bars) and following 4-hours of food withdrawal (4 pm to 8 pm; control: grey bars; p53MKO: orange bars) in mice directly after an abbreviated course of short-term (3-day) tamoxifen administration. Quantifications were normalized to total corresponding protein (for p-PDH normalized to total PDH (t-PDH) depicted in main figure 7D) and total protein (TP) loading (n=3-5). Results presented as mean ± SEM and compared by 2-way ANOVA, Mann-Whitney test, or multiple t-tests; * p<0.05, ** p<0.01, *** p<0.001, *** p<0.001.

Figure S9. (**A**) Representative detection (left panel) of protein levels of LC3-II and LC3-I in quadriceps muscle of short-term (3-day) tamoxifen treated control and p53MKO mice in the fed state (control: white bars; p53MKO: blue bars) and following 4-hours of food withdrawal (control: grey bars; p53MKO: dark blue bars) and quantification of LC3II:LC3I ratio normalized to total protein (right panel). (**B**) mRNA expression of *Fbxo32* and *Trim63* in quadriceps muscle under similar conditions. Results presented as mean ± SEM and compared by 2-way ANOVA (*n*=4-5 for A and B); * $p<0.05$, ** $p<0.01$, *** $p<0.001$, **** $p<0.0001$.

Figure S10. (**A**) Circulating free fatty acids (FFA), triglycerides (TG), urea, and (**B**) insulin in control and p53MKO mice in the fed state (control: white bars; p53MKO: blue bars) and following 16-hours of food withdrawal (control: grey bars; p53MKO: dark blue bars) in mice the received the full course of 5-day tamoxifen treatment (n=10-11). (**C**) Circulating free fatty acids (FFA) and triglycerides (TG) in control and p53MKO mice in the fed state (control: white bars; p53MKO: yellow bars) and following 4-hours of food withdrawal (control: grey bars; p53MKO: orange bars) in mice the received the full course of 5-day tamoxifen treatment (n=7-8); * $p<0.05$, ** $p<0.01$, *** $p<0.001$, **** $p<0.0001$.

Figure S11. (**A**) Gene expression of p53 target genes *Cdkn1a*, *Ddit4*, *Lpin1*, and *Sesn2* in wild-type C57BL/6J mice at rest (white bars) and after an acute endurance treadmill run until exhaustion (grey bars). Control (black line) and p53MKO (green line) mice underwent an acute endurance treadmill run in a metabolic chamber, in which gas exchange was measured (n=3-5). (**B**) Carbohydrate oxidation rate, (**C**) percent carbohydrate oxidation, (**D**) lipid oxidation rate, (**E**) percent lipid oxidation, (**F**) respiratory exchange ratio (RER), (**G**) oxygen consumption (VO2), (**H**) carbon dioxide expiration (VCO2) in control and p53MKO animals (n=4). The dashed line in panel F represents the point at which fuel utilization is equal between carbohydrate and lipid substrates. Results presented as mean ± SEM and compared by Mann-Whitney test or multiple t-tests; * $p<0.05$, ** $p<0.01$, *** $p<0.001$.

Figure S12. (**A**) Plasma metabolites and (**B**) insulin hormone in control (white bars) and p53MKO (green bars) animals after acute endurance run. Gene expression of genes regulating fatty acid oxidation in (**C**) quadriceps and (**D**) gastrocnemius muscle from control and p53MKO mice. (**E**) Normalized gene counts

(log2) of fatty acid metabolism genes from RNAseq analysis of quadriceps muscle. Results presented as mean ± SEM and compared by Mann-Whitney test (n=3–4 in all panels).

Table legends

Table S1. Transcriptomic analysis of upregulated KEGG pathways in quadriceps muscle by fasting (16 hours) compared with fed mice.

Table S2. Transcriptomic analysis of downregulated KEGG pathways in quadriceps muscle by fasting (16 hours) compared with fed mice.

Table S3. Transcriptomic analysis of upregulated genes (upregulated by fasting [16 hours] compared with fed control mice) appearing in KEGG pathway analysis of quadriceps muscle.

Table S4. Transcriptomic analysis of downregulated genes (downregulated by fasting [16 hours] compared with fed control mice) appearing in KEGG pathway analysis of quadriceps muscle.

Table S5. Transcriptomic analysis of upregulated KEGG pathways in quadriceps muscle of p53MKO mice compared to control mice with *ad libitum* feeding.

Table S6. Transcriptomic analysis of downregulated KEGG pathways in quadriceps muscle of p53MKO mice compared to control mice with *ad libitum* feeding.

Table S7. Transcriptomic analysis of upregulated gene ontology terms (biological processes) in quadriceps muscle of p53MKO mice compared to control mice with *ad libitum* feeding.

Table S8. Transcriptomic analysis of downregulated gene ontology terms (biological processes) in quadriceps muscle of p53MKO mice compared to control mice with *ad libitum* feeding.

Table S9. Transcriptomic analysis of upregulated KEGG pathways in quadriceps muscle of p53MKO mice compared to control mice, after acute endurance exercise.

Table S10. Transcriptomic analysis of downregulated KEGG pathways in quadriceps muscle of p53MKO mice compared to control mice, after acute endurance exercise.

Table S11. Primer sequences for qPCR analyses.

Supplementary Methods

For murine genotyping, the following primers were used:

**Oligonucleotides for genotyping**		
**Description**	**Source**	**Identifier #**
Genotyping for *CreERT2* allele, (5'→3') Forward primer: CATTTGGGCCAGCTAAACAT, Reverse primer: CCCGGCAAAACAGGTAGTTA	This paper	NA
Genotyping for *Trp53*$^{lox/lox}$ allele, (5'→3') Forward primer: CTACCTGAAGACCAAGAAGG, Reverse primer: TGGAGGATATGGACCCTATG	This paper	NA

**Software and algorithms used**		
**Description**	**Source**	**Identifier #**
ImageJ	Fiji	RRID:SCR_003070
GraphPad Prism (v8.1.2)	1992-2020 GraphPad Software, LLC	RRID:SCR_002798
DAVID Bioinformatics Resource (v6.8)	NA	RRID:SCR_001881
DESeq2 (v.28.1)	NA	RRID:SCR_015687
R (v3.5.2)	NA	RRID:SCR_001905
HISAT v2.0.4	NA	RRID:SCR_015530
Bowtie2 v2.2.5	NA	RRID:SCR_016368
RSEM (v1.2.12)	NA	RRID:SCR_013027
Bio-Rad CFX Manager (v3.1)	Bio-Rad	https://www.bio-rad.com/
Fusion FX Edge	Peqlab Biotechnology	NA
Odyssey CLx imaging system	Li-cor	https://www.licor.com/
PhenoMaster	TSE Systems	https://www.tse-systems.com/product-details/phenomaster/

Madison-Qingdao Metabolomics Consortium Database	NA	[1]
Bruker Topspin (v3.1) NMR Software	Bruker	https://www.bruker.com/en/products-and-solutions/mr/nmr-software.html
Mnova (v10.0)	Mestrelab Research	https://mestrelab.com/download_file/mnova-10-0-0/
Cellpose	NA	RRID:SCR_021716
BZ 9000 Fluorescence Microscope	Keyence	RRID:SCR_015486

Animal models

All mice were maintained at 22°C under regular 12-hour-light/12-hour-dark cycles. C57BL/6J wild-type mice were maintained within our animal facility (Strain Code: 632; Charles River, Sulzfeld, Germany). Experiments using C57BL/6J wild-type, male mice were performed at 9 weeks of age. For acute, myofiber-specific inactivation of *Trp53*, mice carrying a heterozygous allele expressing a tamoxifen-inducible version of Cre-recombinase under control of the human alpha-skeletal actin promoter (Strain: Tg(ACTA1-cre/Esr1*)2Kesr/J; Stock No: 025750; The Jackson Laboratory, Bar Harbor, ME, USA) were intercrossed with animals carrying lox-P flanked alleles of the *Trp53* gene (Strain: FVB.129P2-Trp53tm1Brn/Nci; Stock No. 01XC2, The National Cancer Institute (NCI) Mouse Repository). Strains were previously backcrossed to C57BL/6J mice for more than 10 generations to obtain a homogenous genetic background. Animals homozygous for the lox-P flanked allele of *Trp53* and heterozygous for the CreERT2-allele were designated as allele carriers to generate a muscle-specific knockout for p53 (p53mKO; genotype: $Trp53^{lox/lox}/CreERT2^{+/WT}$) and littermates homozygous for the lox-P flanked allele of *Trp53* and negative for the CreERT2-allele were considered control mice (LOX; genotype: $Trp53^{lox/lox}/CreERT2^{WT/WT}$). Prior to experimental procedures, *LOX and* p53MKO mice were maintained on a low-phytoestrogen diet (ssniff *Spezialdiäten* GmbH, Soest, Germany) for at least 5 weeks. Tamoxifen (Molekula, Munich, Germany; 2 mg), dissolved in 90% peanut oil (Sigma-Aldrich, Darmstadt, Germany) /10% ethanol, was administered daily as intraperitoneal injections for 5 consecutive days, except when indicated otherwise in the respective experiments, in a total volume of 100 µl per injection,

and animals were sacrificed 2 days after the end of tamoxifen treatments. Body composition was measured by small animal nuclear magnetic resonance spectroscopy (EchoMRI, Echo Medical Systems, Houston, TX, USA). For food withdrawal experiments, food was removed in the afternoon, around 4 pm, and animals were killed the next morning after 16 hours of fasting. In the short-term experiment, mice were killed in *ad libitum* fed state or after being fasted for four hours, from 4 pm to 8 pm before tissue collection.

Transcriptome analysis by RNA sequencing

For library preparation and sequencing, total RNA was isolated from quadriceps muscles collected from ad libitum fed, 16-hour fasted, or acutely endurance exercised mice (n = 3–5). Agilent 2100 Bio analyzer (Agilent RNA 6000 Nano Kit) was used to check for total RNA quality of the samples. RNA-sequencing was performed using the BGISEQ-500 platform (BGI Genomics, Guangdong, China). For data processing and analysis, sequenced reads were filtered by applying SOAPnuke v1.5.2. Reads containing adapter sequences or more than 10% of unknown bases were filtered out. The clean reads were mapped to a reference genome (Genome Reference Consortium Mouse Build 38 mm10) using the alignment tools HISAT v2.0.4 and Bowtie2 v2.2.5. Expression levels were quantified with RSEM v1.2.12. For analysis of two groups, (raw) count data was normalized using DESeq2 (v.28.1) internal normalization, followed by pairwise differential expression analysis [2], with Wald statistics for calculation of P-value with adjustment for multiple testing using the Benjamini-Hochberg method [3, 4]. For analyses of more than two groups, FPKM values were normalized by quantile normalization [5]. For subsequent statistical analysis, P-value calculation for differential expression of genes between the groups was performed using Welch's t-test, with additional Benjamini-Hochberg adjustment [3, 4] using R (v3.5.2) [6]. In all cases, differentially expressed genes were selected at a P-value cut-off of < 0.05. Functional analysis was performed using the online Database for Annotation, Visualization and Integrated Discovery (DAVID Bioinformatics Resource 6.8) tool [7, 8]. Principle component analysis (PCA) and hierarchical clustering were performed on a subset of 45 differentially expressed genes involved in circadian rhythm, as identified by the functional analysis. The clustering was computed with mean expression values of the conditions, using Euclidean distance and complete linkage, and represented as a dendrogram using R software version 3.5.2 [6].

***In vivo* indirect calorimetry**

Indirect calorimetry experiments were performed with the PhenoMaster/Labmaster System (TSE Systems, Bad Homburg, Germany), which was coupled to analysis of measured locomotor activity using the InfraMot-Activity System (TSE Systems). For the assessment of exercise endurance, animals were prompted to run on a mouse treadmill (Exer 3/6, Columbus Instruments, Ohio, USA) with increasing speed (see supplementary methods for detailed protocol). To determine fuel utilization in running animals, mice were prompted to run on a closed-circuit metabolic treadmill (Columbus Instruments, Columbus, OH, USA). For determination of carbohydrate and lipid oxidation rates, the following calculations were used [9]:

- Carbohydrate oxidation: (4.55 x VCO2) - (3.21 x VO2) x 4
- Lipid oxidation: (1.67 x VO2) - (1.67 x VCO2) x 9

To convert from mass per time to kcal per time units, a factor of 4 and 9 for carbohydrate and lipid oxidation calculation, respectively, was factored [10].

***In vivo* exercise capacity measurements**

<u>Treadmill running protocol</u>

Step	Start Speed (m/min)	End Speed (m/min)	Step	Start Speed (m/min)	End Speed (m/min)
1	0	0	12	28	32
2	0	12	13	32	32
3	12	12	14	32	36
4	12	16	15	36	36
5	16	16	16	36	40
6	16	20	17	40	40
7	20	20	18	40	44
8	20	24	19	44	44
9	24	24	20	44	48
10	24	28	21	48	48
11	28	28			

Prompting was aided with an electric grid, and animals were considered exhausted when resting for ≥5 sec on the grid. Animals were sacrificed 15 min post exercise. For the analysis of fuel selection during exercise on closed-system metabolic treadmill running with indirect calorimetry, mice were prompted to run on a treadmill enclosed in a tight chamber (Columbus Instruments, Ohio, USA). Lipid and carbohydrate oxidation rates (mg / kg BW / min) were assed using the following equations [11, 12]:

- Carbohydrate oxidation: 4.21 · VCO_2 (mL/kg BW/h) – 2.962 · VO_2 (mL/kg BW/h) / 60
- Lipid oxidation: 1.695 · VO_2 (mL/kg BW/h) – 1.701 · VCO_2 (mL/kg BW/h) / 60

To calculate the percentage of non-protein fuel oxidation, RER values between 0.7 and 1.0 were extrapolated to percent of carbohydrate *vs*. lipid oxidation, based on the findings of Lusk, 1923 [11, 13].

<u>Calculation of carbohydrate and lipid oxidation:</u>

**RER**	**CH (%)**	**Lipid (%)**
0.70	0.00	100.00
0.71	1.40	98.60
0.72	4.80	95.20
0.73	8.20	91.80
0.74	11.60	88.40
0.75	15.00	85.00
0.76	18.40	81.60
0.77	21.80	78.20
0.78	25.20	74.80
0.79	28.60	71.40
0.80	32.00	68.00
0.81	35.40	64.60
0.82	38.80	61.20
0.83	42.20	57.80
0.84	45.60	54.40
0.85	49.00	51.00
0.86	55.80	47.60

0.87	52.40	44.20
0.88	59.20	40.80
0.89	62.60	37.40
0.90	66.00	34.00
0.91	69.40	30.60
0.92	72.80	27.20
0.93	76.20	23.80
0.94	79.60	20.40
0.95	83.00	17.00
0.96	86.40	13.60
0.97	89.80	10.20
0.98	93.20	6.80
0.99	96.60	3.40
1.00	100.00	0.00

The crossover point represents the switch from primarily lipid oxidation to carbohydrate oxidation. The time at first sustained crossover was determined as the point at which lipid oxidation was lower than 50% for more than five subsequent intervals (each interval is 30 seconds).

Quantitative real-time PCR

Total RNA was isolated with the RNA MiniPrep Kit (Zymo Research, Freiburg, Germany) for RNA-seq analyses or by the phenol-chloroform method for all other analyses. cDNA synthesis was performed with the Applied Biosystems™ High-Capacity cDNA Reverse Transcription Kit (Thermo Fisher Scientific, Dreieich, Germany) and diluted to 4 ng/µL for quantitative real-time PCR (qPCR). qPCR was performed using the Biorad CFX384 Real-Time System (Biorad, Munich, Germany) with Maxima™ SYBR™ Green/ROX 2x qPCR Master Mix (Thermo Fisher Scientific, Dreieich, Germany). Primer sequences are listed in suppl. Table S11. mRNA expression was calculated relative to the mRNA expression of housekeeper gene *Arbp*.

**Reagents used**		
**Description**	**Source**	**Identifier #**

Pierce BCA protein assay kit	Thermo Fisher Scientific	10741395
Direct-zol RNA Mini Prep Kit	Zymo Research	R2052
Applied Biosystems™ High-Capacity cDNA Reverse Transcription Kit	Thermo Fisher Scientific	10186954
Maxima™ SYBR™ Green/ROX 2x qPCR Master Mix	Thermo Fisher Scientific	10100790

Histology and cross-sectional myofiber area

Quadriceps muscles were dissected and fixed overnight in 4% formaldehyde at 4ºC. Muscle samples were dehydrated and embedded in paraffin. 2 µm sections were cut in the mid-region of the muscle and transferred to glass slides. Sirius red staining was performed on deparaffinized sections. Briefly, slices were incubated with a 0.1% Sirius red solution dissolved in aqueous saturated picric acid for 1 hour, washed in acidified water (0.5% acetic acid), dehydrated and mounted with Fluoromount-G (Thermo Fisher Scientific, Dreieich, Germany). Collagenous components were red-stained to differentiate muscle fibers from the extracellular matrix. Staining was visualized in a BZ900 Fluorescence Microscope (Keyence, Neu-Isenburg, Germany) and CellPose deep-learning algorithm was used to automatically segment individual fibers from regions of dense red extracellular matrix [14] . Fiber size was determined by measuring the cross-sectional area from two different areas of sections per mouse and analyzed with FIJI/ImageJ and plugin LaberlsToROIs, as described before [15].

Proteasome activity (chymotrypsin-like activity)

Proteasome activities were measured as described before [16]. In brief, muscle tissue samples were homogenized in lysis buffer (250 mM sucrose, 25 mM HEPES, 1 mM EDTA, 10 mM magnesium chloride and freshly added 1.7 mM DTT, pH 7.8) using a ball mill (2x2min, 30Hz). The homogenates were then passed 20 times through a 27-gauge needle attached to a 1 ml syringe, followed by 3 times freeze-thaw cycles and centrifugation at 13,400 rpm, for 10 min, 4°C. Supernatants were used for determination of protein (Bradford assay) and proteasome activity. Prior to the activity measurement, all samples were adjusted to 1 mg/ml protein and incubated with proteasome activity incubation buffer (225 mM Tris buffer (pH 7.8), 7.5 mM magnesium acetate, 45 mM potassium chloride, 7.5 mM magnesium chloride and freshly added 1 mM DTT). To measure 20S proteasome activity, ATP was depleted by adding 15 mM 2-deoxyglucose and 0.1 mg/ml hexokinase to the incubation buffer. For measurement of 26S

proteasome activity, further ATP was added to the incubation buffer, final ATP concentration 2 mM. Samples were shaken in the respective incubation buffers for 2 min, 100 rpm, followed by 10 min incubation at room temperature.

Citrate synthase activity

Citrate Synthase (CS) activity was determined spectrometrically by monitoring the reduction of DTNB (5,5′ dithiobis-(2-nitrobenzoic acid) concentrations at 412 nm. CS buffer containing 50 mM Tris, 1 mM EDTA (pH=7.4), and 0.1% Triton X-100 was used to homogenize gastrocnemius muscle tissue followed by a 13,000×g centrifugation for 10 min at 4 °C. An aliquot of 10 µL of 1:10 diluted supernatant was mixed with 215 µL of reaction buffer (100 mM Tris, 1 mM MgCl2, 1 mM EDTA adjusted to pH=8.2, 0.1 M DTNB) and 25 µL of Acetyl-CoA (3.6 mM) into 96-well plate. The reaction was initiated by adding 3 mM Oxaloacetate and the increase in absorbance at 412 nm was measured for 10 min at 37°C. The CS activity was calculated from the slope of the linear portion and normalized to mg tissue. All analyses were measured as triplicates.

Metabolite analyses by NMR spectroscopy

Powdered skeletal muscle tissue (30 mg) was homogenized in ice-cold phosphate-buffered saline (PBS) using the Bullet Blender (Next Advance, NY, USA) and stainless-steel beads (0.9–2 mm diameter blend; Next Advance, NY, USA). Samples were sonicated (UP-200S ultrasonic power device, Hielscher Ultrasound Technology, Teltow, Germany) at 20% amplitude for 6 cycles (cycles at 0.25). 1.2 mL of ice-cold methanol was added, then samples were vortexed and stored at –20 °C for 30 min, followed by centrifugation at 12,000 x g, for 30 min at 4 °C. The supernatants were lyophilized for 2–3 hours without heat using a speed vac pumping unit (PC 3003) and controller (CVC 3000; Vacuubrand, Wertherim, Germany) to evaporate PBS / methanol. Samples were re-dissolved in 500 µL of NMR buffer [0.08 M Na_2HPO_4, 5 mM TSP (3-(trimethylsilyl) propionic acid-2,2,3,3-d_4 sodium salt), 0.04 (w/v) % NaN_3 in D_2O, pH adjusted to 7.4 with 8 M HCl and 5 M NaOH] and transferred to 5 mm NMR tubes. All experiments were carried out on a Bruker Avance Neo 600 MHz spectrometer, with a TXI probe head (Bruker, Rheinstetten, Germany) at 310 K. For ^1H 1D NMR experiments, the 1D Carr-Purcell-Meiboom-Gill pulse sequence with pre-saturation was used (cpmgpr1d; 73728 points in F1; 12019.230 Hz spectral width; 2048 transients; recycle delay 4 sec). Data were processed in Bruker Topspin version 4.0.2 (Bruker Biospin, Rheinstetten, Germany) using one-dimensional exponential window multiplication of the FID,

Fourier transformation, and phase correction. NMR data were then imported to Matlab2014b. TSP was used as internal standard for chemical shift referencing (set to 0 ppm). Regions around the water, TSP, and methanol signals were excluded, NMR spectra were aligned, and a probabilistic quotient normalization was performed. Reference chemical shifts and assignment for metabolites were obtained from the Human Metabolome Database (HMDB) [1] and Chenomx NMR Suite 7.6 (Chenomx Inc., Edmonton, AB, Canada). Quantification of metabolites was carried out by signal integration of normalized spectra.

Plasma analyses

Blood was collected from the *vena facialis* or heart (for corticosteroid hormone analysis) and centrifuged for 10 min at 6,200 x g (4°C). Plasma metabolites were measured using the Roche COBAS MIRA S (Roche Diagnostics, Mannheim, Germany). Glucose, lactate, triglycerides, cholesterol (all from: Axon Lab AG, Reichenbach, Germany) and free fatty acids (Fuji Film Healthcare Solutions, Lexington, MA, USA) were measured using kits according the manufacturer's instructions. Circulating alanine was measured using a colorimetric assay kit (Sigma Aldrich, Darmstadt, Germany) following the manufacturer instructions with a prior plasma deproteinization step using a 10 kDa MWCO spin filter (Merck Millipore, Darmstadt, Germany). Insulin, corticosterone and cortisol were measured by enzyme-linked immunosorbent assay (Insulin: Crystal Chem, Elk Grove Village, IL, USA; Corticosterone: Arbor Assays, Ann Arbor, MI, USA; Cortisol: BioCat, Heidelberg, Germany). Commercial assays used were:

Description	Source	Identifier #
Insulin hormone assay	Crystal Chem	90010
Cortisol hormone assay	BioCat	K7430-100-BV
Corticosterone hormone assay	Arbor Assays	K014-H1
Starch hydrolysis kit	R-Biopharm AG	10207748035
Alanine assay kit	Sigma Aldrich	MAK001-1KT
10 kDa MWCO spin filter	Merck Millipore	MRCPRT01

Liver and muscle glycogen analyses

Liver and quadriceps muscle tissue were ground to powder and homogenized in 0.1 M NaOH with zirconium beads using a SpeedMill Plus homogenizer (Analytik Jena AG, Jena, Germany). Following short centrifugation, samples were heated in a heated shaker for 45 min (70°C; 1000 rpm), followed by

mixing and centrifugation at 12,400 x g for 45 min (4°C). Supernatants were centrifuged again at 12,400 x g for 30 min (4°C). Glycogen was determined using a starch hydrolysis kit (R-Biopharm AG, Pfungstadt, Germany) using the manufacturer's instructions.

The method is for glycogen quantification was based on the following reactions:

- Glycogen/starch + H2O → n D-glucose (catalyzed by amyloglucosidase [AGS])
- D-glucose + ATP → glucose-6-phosphate (G-6-P) + ADP (catalyzed by hexokinase)
- G-6-P + NADP+ → D-gluconate-6-phosphate + NADPH + H+ (catalyzed by G-6-P dehydrogenase)

To differentiate between free glucose present in the samples and glucose released by the hydrolysis of glycogen chains, samples and standards were split into two samples sets: A (+ AGS enzyme) and B (– AGS enzyme). Following a 15 min incubation at 60°C on a heated shaker and subsequent centrifugation at 23,1000 x g for 10 min, the supernatant was pipetted into 96-well plates and an ATP/NADP mix was added. Plates were mixed briefly and read at 340 nm with a microplate reader (Synergy H1, BioTek, Vermont, USA). Finally, hexokinase and G-6-P dehydrogenase enzymes were added to the wells, followed by 15 min shaking and absorbance reading at 340 nm, representing the level of NADPH in each sample. To determine the level of glucose present in glycogen form only, readings from sample set B were subtracted from sample set A, and data were normalized to protein levels, measured with a BCA protein assay kit (Thermo Fisher Scientific, Dreieich, Germany).

Liver triglyceride analyses

Frozen liver tissue was ground to powder in liquid nitrogen and was subsequently homogenized in HB buffer (10 mM NaH_2PO_4, 1 mM EDTA, 1% polyoxyethylene [10] tridecyl ether [v/v], pH 7.4) in a bullet blender using stainless steel mixed-size beads (0.9–2 mm diameter, Next Advance, New York) for 5 min. Samples were centrifuged for 30 min at 18,000 x g (4°C), and the supernatant was incubated in a heated shaker (Eppendorf, Hamburg, Germany) for 5 min at 70°C. Samples were cooled on ice and spun again at 18,000 x g for 30 min (4°C). The supernatant was measured for triglycerides using the Roche COBAS MIRA S instrument (Roche Diagnostics, Mannheim, Germany) and triglyceride assay kit according to manufactures instructions (Axon Lab AG, Reichenbach, Germany).

Pyruvate dehydrogenase (PDH) activity

The PDH assay was performed according to the manufacturer's instructions (Order-ID K679-100, BioVision, Milpitas, CA, USA).

Immunoblotting

Tissues were homogenized in RIPA buffer supplemented with protease inhibitor cocktail (1:100; Sigma Aldrich, Darmstadt, Germany) and phosphatase inhibitor cocktails 2 and 3 (1:100, Sigma Aldrich, Darmstadt, Germany) using a hand-held stick blender (VWR International GmbH, Dresden, Germany), on ice, followed by sonication using a UP-200S ultrasonic power device (Hielscher Ultrasound Technology, Teltow, Germany) for 6 cycles (0.25) at 20% amplitude, and centrifugation at 12,000 x g for 15 min at 4°C. Protein concentrations were determined using a BCA protein assay kit (Thermo Fisher Scientific, Dreieich, Germany) using the manufacturer's protocol. Protein lysates were diluted to For OXPHOS-complexes, muscle tissue was homogenized in Citrate Synthase (CS, see corresponding method description) buffer and protein lysates were diluted in 1 µg/µL Laemmli buffer with β-mercaptoethanol (1:1000), then boiled at 37°C for 5 min. 1 µg/µL in Laemmli buffer supplemented with 1:1000 β-mercaptoethanol, then boiled at 95°C for 5 min. Antibodies used are listed below in resources table. All proteins were normalized to total protein for quantification. Following SDS-PAGE, proteins were transferred onto PVDF membranes (Merck Millipore, Darmstadt, Germany). Membranes were stained with reversible protein stain (Thermo Fisher Scientific, Dreieich, Germany, or Li-cor Biosciences, Bad Homburg, Germany) and quantified using the Li-cor immunofluorescent detection system (Li-cor Biosciences, Bad Homburg, Germany) or enhanced chemiluminescence (ECL) with the Amersham ECL Western Blotting Detection Reagent (GE Healthcare, Chicago, IL, USA). Detection was performed using the Quantum ST4 (Peqlab Biotechnology, Erlangen, Germany) or the Odyssey CLx imaging system (Li-cor Biosciences, Bad Homburg, Germany). Quantification was performed using Image J software (U.S. National Institutes of Health, Bethesda, MD, USA; [17]) and normalized to total protein levels, unless otherwise stated, and each band signal was normalized to the maximal band signal within each blot prior to pooling.

**Antibodies used**		
**Description**	**Source**	**Identifier #**
Anti-CaMKII (pan) Rabbit 1:1000	Cell Signaling Technology	4436S

Anti-LC3B Rabbit 1:1000	Cell Signaling Technology	2775S
Anti-Glycogen synthase Rabbit 1:1000	Cell Signaling Technology	3886
Anti-GSK-3-β Rabbit 1:1000	Cell Signaling Technology	9315
Anti-K48-linkage specific polyubiquitin-HRP 1:1000	Cell Signaling Technology	12805S
Anti-K63-linkage specific polyubiquitin 1:1000	Cell Signaling Technology	5621S
Anti-phospho-GSK-3-β (Ser9) Rabbit 1:1000	Cell Signaling Technology	5558S
Anti-phospho-CaMKII (Thr286) Rabbit 1:1000	Cell Signaling Technology	12716T
Anti-PDH E1-α Mouse 1:1000	Abcam	ab110334
Anti-phospho-GS (Ser641) Rabbit 1:1000	Cell Signaling Technology	3891
Anti-phospho-PDH E1-α (Ser232) Rabbit 1:1000	Abcept	AP1063
Anti-phospho- PDH E1-α (Ser293) Rabbit 1:1000	Abcam	ab92696
Anti-phospho- PDH E1-α (Ser300) Rabbit 1:1000	Sigma Aldrich / Merck	ABS194
Anti-PDK1 Mouse 1:1000	Abcam	Ab110025
Anti-PDK2 Rabbit 1:1000	Abcam	Ab68164
Anti-PDK4 Rabbit 1:1000	Novus Bio	NBP1-07047
Anti-total OXPHOS-cocktail Mouse 1:1000	Abcam	Ab110413
Anti-PCX Rabbit 1:1000	Novus Bio	NBP1-49536
Anti-PYGM Rabbit 1:1000	Abcam	ab231963
IRDye 680RD donkey anti-mouse 1:15,000 secondary antibody Li-cor	Li-cor	925-68070
IRDye 800RD donkey anti-rabbit 1:15,000 secondary antibody Li-cor	Li-cor	925-32213

References

1. Wishart DS, Feunang YD, Marcu A, Guo AC, Liang K, Vazquez-Fresno R, et al. HMDB 4.0: the human metabolome database for 2018. Nucleic Acids Res. 2018;46:D608-D17.
2. Love MI, Huber W, Anders S. Moderated estimation of fold change and dispersion for RNA-seq data with DESeq2. Genome Biol. 2014;15:550.
3. Benjamini Y, Hochberg Y. Controlling the false discovery rate: a practical and powerful approach to multiple testing. Journal of the Royal statistical society: series B (Methodological). 1995;57:289-300.

4. Benjamini Y, Yekutieli D. Resampling-based false discovery rate controlling multiple test procedures for correlated test statistics. Journal of Statistical Planning and Inference. 1999;82:171-96.
5. Bolstad BM, Irizarry RA, Astrand M, Speed TP. A comparison of normalization methods for high density oligonucleotide array data based on variance and bias. Bioinformatics. 2003;19:185-93.
6. Computing RALaEfS. R Development Core Team . R Foundation for Statistical Computing. 2018.
7. Huang da W, Sherman BT, Lempicki RA. Systematic and integrative analysis of large gene lists using DAVID bioinformatics resources. Nat Protoc. 2009;4:44-57.
8. Huang da W, Sherman BT, Lempicki RA. Bioinformatics enrichment tools: paths toward the comprehensive functional analysis of large gene lists. Nucleic Acids Res. 2009;37:1-13.
9. Frayn KN. Calculation of substrate oxidation rates in vivo from gaseous exchange. J Appl Physiol Respir Environ Exerc Physiol. 1983;55:628-34.
10. Schilperoort M, van Dam AD, Hoeke G, Shabalina IG, Okolo A, Hanyaloglu AC, et al. The GPR120 agonist TUG-891 promotes metabolic health by stimulating mitochondrial respiration in brown fat. EMBO Mol Med. 2018;10.
11. Peronnet F, Massicotte D. Table of nonprotein respiratory quotient: an update. Can J Sport Sci. 1991;16:23-9.
12. Petrosino JM, Heiss VJ, Maurya SK, Kalyanasundaram A, Periasamy M, LaFountain RA, et al. Graded Maximal Exercise Testing to Assess Mouse Cardio-Metabolic Phenotypes. PLoS One. 2016;11:e0148010.
13. Lusk G. The elements of the science of nutrition. 3 ed. Philadelphia: W.B. Saunders Company; 1923.
14. Stringer C, Wang T, Michaelos M, Pachitariu M. Cellpose: a generalist algorithm for cellular segmentation. Nat Methods. 2021;18:100-6.
15. Waisman A, Norris AM, Elias Costa M, Kopinke D. Automatic and unbiased segmentation and quantification of myofibers in skeletal muscle. Sci Rep. 2021;11:11793.
16. Reeg S, Jung T, Castro JP, Davies KJA, Henze A, Grune T. The molecular chaperone Hsp70 promotes the proteolytic removal of oxidatively damaged proteins by the proteasome. Free Radic Biol Med. 2016;99:153-66.
17. Schneider CA, Rasband WS, Eliceiri KW. NIH Image to ImageJ: 25 years of image analysis. Nat Methods. 2012;9:671-5.
